# Importance of scale in seascape habitat suitability modeling: Predicting species distributions in changing ecosystems

**DOI:** 10.1002/eap.70318

**Published:** 2026-11-04

**Authors:** Herbert Leavitt, Alexander Thomas, James A. Nelson

**Affiliations:** ^1^ Department of Marine Sciences University of Georgia Athens Georgia USA; ^2^ Present address: Department of Earth and Environment, Institute of Environment Florida International University Miami Florida USA

**Keywords:** climate change, drone imagery, habitat suitability models, landscape change, remote sensing, satellite imagery, spatial scale, species distribution models

## Abstract

Habitat suitability models (HSMs) use landscape metrics to describe patterns in species distributions, quantify the success of habitat restoration, and model species responses to change. Within a particular level of organization, the spatial scale of effect at which habitat is integrated and incorporated into HSMs can influence model performance. However, scale optimization can be time consuming and requires high‐quality data across spatial scales. As such, it is not widely applied in suitability models and is particularly rare in marine studies. In coastal and marine landscapes, or seascapes, rapidly changing seascape metrics and environmental contexts can disrupt known species‐seascape relationships and complicate evaluations of habitat quality. Studying the effect of scale on species suitability models in these systems allows for greater predictive power and better understanding of the multiscale processes that drive species distributions. We use quantitative species abundance from a Louisiana Gulf coast estuary along with both drone and satellite‐derived habitat data to model species‐habitat relationships across a range of measurement scales. Using this approach, we identify optimal measurement scales for population‐level HSMs for *Penaeus setiferus* (white shrimp) and *Callinectes sapidus* (blue crabs) and identify key species‐habitat relationships. Within the range of scales considered (20–600‐m radius), blue crab HSMs had the highest predictive power at 150 m. White shrimp showed high predictive power at both 120 m and 300 m in drone and satellite‐based analyses, respectively. Our models show that white shrimp have a positive association with *Avicennia germinans* (black mangroves), a range‐expanding foundation species rapidly encroaching onto the *Spartina alterniflora* marshes in Louisiana. This approach generates predictive HSMs for two key fisheries species on the Louisiana coast and helps define their relationship to ongoing changes in their habitat. Future seascape suitability studies should test across spatial scales to optimize the predictive power of their models.

## INTRODUCTION

Ecologists and managers use habitat suitability models (HSMs) to quantitatively determine the capacity of a particular habitat to support a given species. These models can be applied across scales, from geographical species ranges to patch and microhabitat use (Meyer & Thuiller, 2006). The spatial extent, resolution, and features included in these models depend on the goals of the model, biology of the species, and availability of data, typically incorporating environmental, habitat, or resource‐related variables. In conservation and restoration, HSMs are increasingly used to enhance landscapes for species of conservation concern (Guisan et al., 2013; Sofaer et al., 2019).

No matter the goal of the HSM, a critical assumption of these models is the scale at which a species is likely to interact with its environment. Habitat selection occurs within a hierarchy of organizational levels, from individual movements to populations to species ranges. Scales of effect can differ depending on the response measured, and some of these responses are sensitive to processes occurring on other organizational levels (Martin, 2018; Miguet et al., 2016). For example, fecundity may have a smaller scale of effect than occurrence, and occurrence patterns might differ when an organism is at the very edge of their geographic range. Identifying reliable scales of effect for habitat suitability studies can be challenging, but the scale of measurement chosen for a given study can greatly affect the predictions of the model (Holland et al., 2004; Meyer et al., 2002; Meyer & Thuiller, 2006). As such, scale optimization is an ongoing challenge in landscape ecology. While there is widespread recognition that multiscale modeling is a valuable tool for understanding ecological relationships, most studies either do not address multiple spatial scales or use a potentially oversimplified framework to select optimized scales (McGarigal et al., 2016; Stuber & Gruber, 2020).

Recent advances in high‐resolution remote sensing and statistical modeling provide new opportunities to evaluate species‐habitat relationships across multiple spatial scales. Satellite and drone imagery allow traditional landscape metrics, such as edge density or land‐to‐water ratio, to be calculated at resolutions that can approximate how individuals experience and interact with their environment. These advancements in data collection have been accompanied by parallel developments in statistical methods that identify which variables and spatial scales drive habitat suitability. This allows researchers to move beyond a priori assumptions about relevant landscape scales and objectively identify a scale that is optimized for predicting their response of interest at the level of organization required by available data or scientific questions. This type of multiscale habitat modeling has been applied extensively in terrestrial systems but remains less common in seascape studies (Boström et al., 2011; Stuber & Gruber, 2020). Seascape ecology itself is a young field, and many of the tenets of landscape ecology remain untested or unapplied in marine settings (Pittman, 2017). Seascape studies that have implemented multiscale analyses show that scale‐dependent patterns influence dynamics of seascape flora and fauna (Kendall et al., 2011; Rodemann et al., 2025; Santos et al., 2011).

Coastal marsh ecosystems are changing rapidly due to climate change and are an ideal test case for understanding the effects of scale and change on HSMs (Harris et al., 2021). Coastal marshes provide a suite of essential services such as carbon storage, coastal protection, and biodiversity support (Barbier et al., 2011) and are undergoing rapid, unprecedented change in habitat composition and seascape structure (Liu et al., 2007; Pittman, 2017). In response to dramatic habitat loss, restoration projects frequently aim to restore lost habitat and recover ecological functions like coastal protection and fisheries habitat. Predicting the value of a project for fisheries species requires accurate HSMs that can model on the scales relevant to project implementation. These smaller scales can be challenging to work with because they require both high‐resolution data and good estimates of relevant scale for small but mobile fisheries species. Additional challenges arise in rapidly changing environments, where shifting conditions may be substantially altering known species‐habitat relationships (Kimball & Eash‐Loucks, 2021; Lesser et al., 2021; Scheffel et al., 2018).

In some of the most vulnerable parts of southern Louisiana, the last two decades have seen a 30% loss in emergent marshes, and a 570% increase in areal cover of black mangroves (*Avicennia germinans*), a foundation species that has expanded its northern range due to decreased frequency of hard freeze events in the region (Comeaux et al., 2012). As mangroves expand into the northern Gulf, they replace the dominant marsh grass foundation species like *Spartina alterniflora*. Mangrove encroachment has not yet been incorporated into habitat suitability assessments for Gulf coast aquatic fauna on large scales for management purposes (Lindquist et al., 2022). However, differences in habitat morphology and trophic interactions observed in *Spartina* and mangrove habitats suggest that they could alter the ecology of coastal saltmarshes by changing resource availability, habitat quality, and predation rates (Glazner et al., 2020; Goeke et al., 2024; Harris et al., 2021; Nelson, Lesser, et al., 2019). As the landscape in the northern gulf changes, managers rely on HSMs to predict how restoration projects, climate change, or development decisions could affect the suitability of the habitat for key species. The Louisiana coastline supports several major commercial fisheries, including blue crab (*Callinectes sapidus*) and white shrimp (*Penaeus setiferus*) that rely on coastal marshes as nursery habitat. Although coarse‐grained models of these species that predict habitat suitability across a large region of coastline (Lindquist et al., 2022) exist, much less is known about fine‐scale, abundance‐based habitat suitability, which can change on spatial scales <1000 m. Models with higher spatial resolution are valuable in that they can operate on the scales that match heterogeneity in seascapes, allowing models to detect the key dynamics that drive habitat use rather than obscuring causal relationships by integrating over meaningful variation. There are many scenarios where these small‐scale dynamics can be of greater use to managers planning restoration or conservation than kilometer scale averages (Rozas et al., 2005). Even as we define the relevant drivers of habitat use, new stressors like mangrove encroachment could be altering the nature and scale of species‐seascape relationships in this rapidly changing landscape.

Scale‐optimized analyses generate more accurate predictions and express ecological patterns in greater detail than single‐scale analyses. Suitability models describing species‐seascape relationships at multiple scales are not only more accurate; the size of a variable's scales of effect also offers insight into the underlying ecology of the species and its interaction with the landscape. We expect certain spatial scales will be better suited for predicting species abundance, and that models including these scales will perform meaningfully better than models at other comparable spatial scales. After determining the scale with the greatest predictive power for abundance of species, we will explore how species respond to habitat metrics at that scale, with an emphasis on metrics like mangrove cover and land‐to‐water ratio, which are known to be rapidly changing in this region.

## METHODS

In this study, we apply a single‐level, multiscale (McGarigal et al., 2016) approach to modeling habitat suitability using abundance data along with satellite‐ and drone‐based aerial imagery. Specifically, we model abundances of common marsh edge associated consumers, white shrimp, blue crab, darter goby, and grass shrimp, to established landscape metrics, such as marsh edge habitat, land‐water ratio, and percent mangrove cover (Armitage et al., 2021; Birdsong, 2004; Jerabek et al., 2017). These species are all mobile nekton, meaning they are strong‐swimming consumers that are capable of foraging over a wide area and selecting for suitable habitat. Our goal is to evaluate if these traditional landscape metrics remain robust predictors of habitat suitability and to determine what spatial scales provide the best predictive power for HSMs of these species. Using abundance as a response variable in these models provides additional information on possible habitat quality and may signal early changes in the species community as the seascape changes (Van Couwenberghe et al., 2013; Waldock et al., 2022).

### Field sampling and sample processing

We collected samples from 52 sites near Port Fourchon, LA, in September 2022. Sites were selected prior to sampling using the package MBHdesign (Foster, 2020) in R (R Core Team, 2022) to select sites in a stratified, spatially balanced quasi‐random design. This method allows for sampling points to be distributed more evenly in space while remaining effectively random. Stratified sampling selected 20 sites with <25%, 25%–75%, and >75% mangrove cover within a 200‐m radius, corresponding to the approximate foraging range of white shrimp (Minello & Rozas, 2002). We collected aquatic fauna from on or adjacent to the marsh edge at each site using a drop sampler with an area of 1.2 m^2^. Once the area was fully enclosed, we used a suction pump with a 1‐mm mesh screen over the outflow to remove water within the ring while capturing small organisms. Remaining organisms were collected with dip nets or by hand and euthanized. We sorted field collections by taxonomic groups and recorded the abundance captured at each site. In the following analysis, we isolate the four most commonly occurring species, which comprised more than 80% of all observations. This included commercially important fisheries species blue crab (*Callinectes sapidus*) and white shrimp (*Penaeus setiferus*) along with grass shrimp (*Palaemonetes* sp.) and darter gobies (*Ctenogobius boleosoma*). The number of individuals of a given species within each drop sample represented a catch per unit effort, which was used as the response variable in the HSMs.

### Habitat cover

We estimated habitat cover by classifying 2022 satellite imagery in ArcGIS Pro. Images of the entire port were selected from Google Earth Engine, georeferenced, and clipped to the study area with a resolution of 2.8 × 3.3 m/pixel. Using ArcGIS Pro's Image Classification Tool, we segmented the satellite image into habitat types based on supervised training data provided by drawing polygons around *Spartina* marsh, mangroves, water, and artificial structures. A support vector machine algorithm classified the image (Devadas et al., 2012; Sohl et al., 2024), with manual corrections applied as necessary.

For scales between 20 and 150 m, we used drone‐based imagery to quantify the habitat metrics. We used a Quantum Systems Trinity F90^+^ fixed‐wing mapping drone equipped with a MicaSense RedEdge‐MX Dual Camera Imaging System with a resolution of 8.37 cm/pixel. We generated orthomosaics using OpenODM (OpenDroneMap Authors, 2020) on the SAPELO2 high‐performance computing cluster at the Georgia Advanced Computing Resource Center (GACRC) and classified the habitat using Trimble eCognition (Trimble Geospatial, 2024) on SAPELO2. eCognition operates by allowing the user to create rulesets that classify objects based on multiple spectral bands. Orthomosaics were segmented into similar objects and then classified using a Nearest Neighbor Classification algorithm (Zhao et al., 2020). Manual rulesets were then applied to correct misspecified objects. Satellite and drone map agreement was compared using confusion matrix analysis (Appendix S1: Table S1). Producer accuracy, which is a proportion of drone‐based pixels that were correctly classified by the satellite map, was 0.78 for “Water” and 0.64 for both “Spartina” and “Mangrove”. Considering that differences in map resolution inherently inflate disagreement, these values are within acceptable limits of accuracy.

To determine the best scale for modeling small consumer abundance, we split our analysis into two spatial extents that use drone‐based and satellite‐based data. For satellite‐based data, we used 9 candidate scales of effect: 70, 100, 150, 200, 250, 300, 400, 500, and 600 m (Figure 1). Early analyses included 700, 800, 900, and 1000 m, but these candidate scales had very low predictive power in preliminary results and were removed for computational efficiency. We chose not to include smaller spatial scales for the satellite‐scale analysis because comparison with drone‐derived landscape metrics suggested that inaccuracies in the more coarse‐grained satellite data exacerbated differences between drone‐derived and satellite‐derived landscape metrics at smaller scales (Appendix S1: Figure S1). See Figure 2 for a visual comparison of drone and satellite data. For drone‐based data, we used seven candidate scales: 20, 30, 50, 70, 100, 120, and 150 m. This made for a total of 16 candidate scales across both drone and satellite–based analyses. Larger scales were not possible for drone‐based data due to limitations on the feasible spatial extent captured by drone imagery.

**FIGURE 1:**
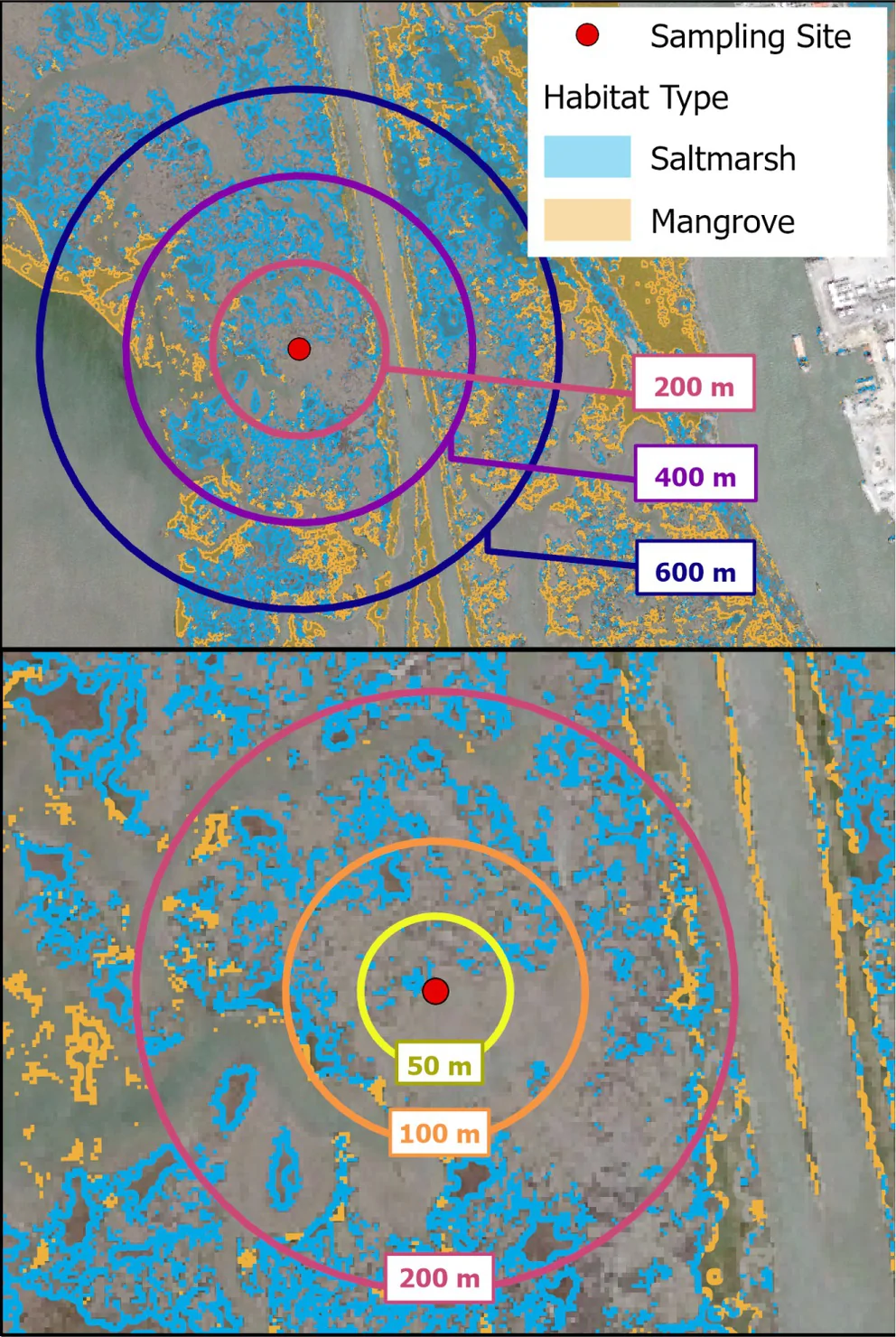
Habitat classification of satellite imagery at a 600‐m buffer (top panel) and a 200‐m buffer (bottom panel) at the same sampling site. The highlighted habitat area indicates the land within 5 m of the water's edge. Since estuarine aquatic fauna interact most strongly with the marsh edge, we considered several edge distances in our models. By running habitat models using measurement scales from 20 to 600 m, we capture highly localized habitat structure out to broad scale landscape patterns.

**FIGURE 2:**
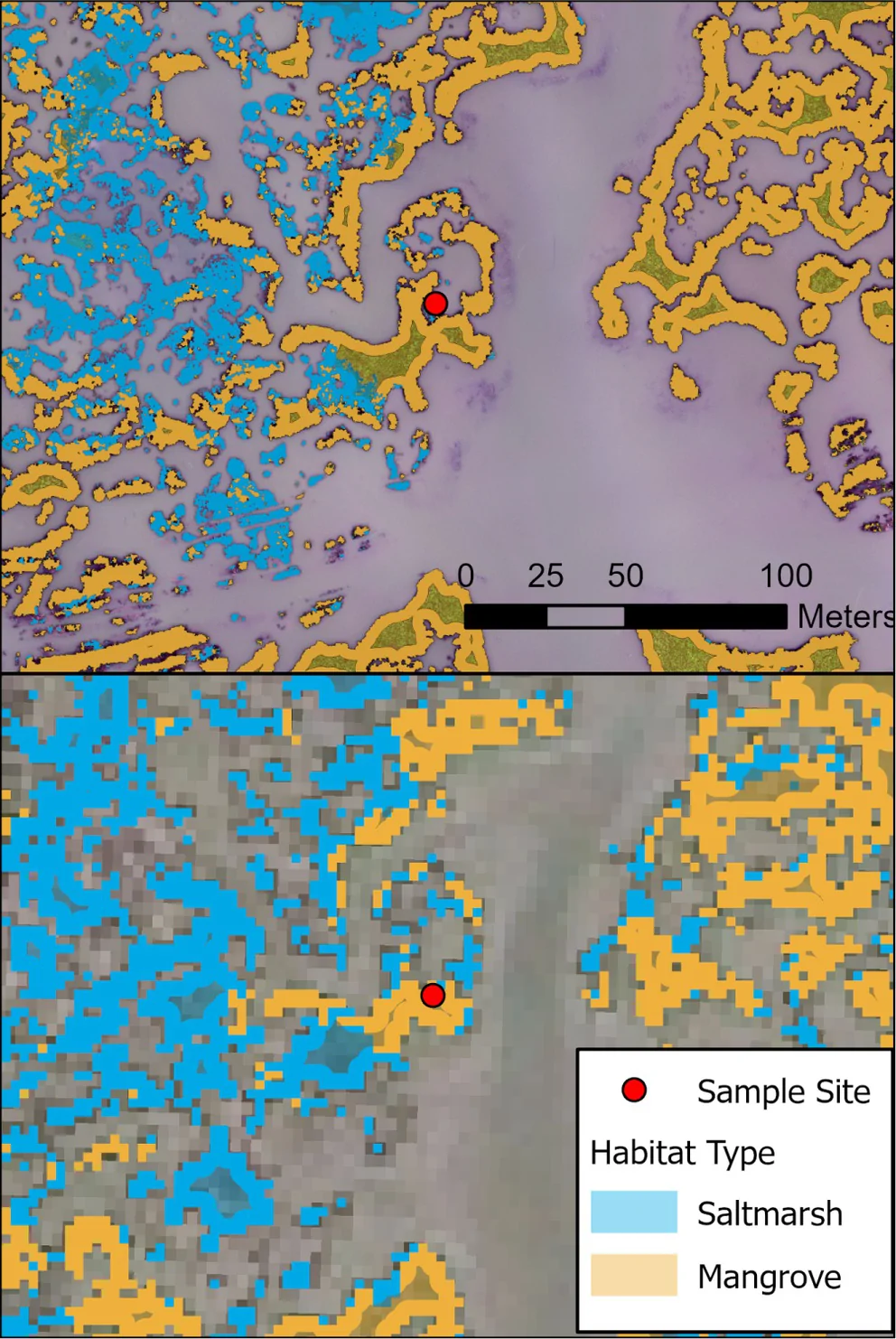
Habitat classifications using satellite‐based data (top panel) and drone‐based data (bottom panel). Maps are scaled to roughly encompass maximum measurement scale for the drone‐based analysis of 150 m. Satellite data could not capture fine details in the landscape structure, making it a poor choice for calculating landscape metrics at small scales. Drone‐derived data are highly accurate but is much more limited in the spatial extent of coverage.

In estuaries, mobile aquatic consumers like the focal species in this study frequently take advantage of production on the marsh surface when it is accessible at high tide (Hollingsworth & Connolly, 2006; Nelson, Johnson, et al., 2019). Minello and Rozas (2002) found that the abundance of numerous marsh species on the marsh was at its maximum on the marsh platform within 1 m of the water's edge and dropped off quickly to nearly zero at 10 m from the water's edge. Nekton rarely interact with the marsh interior; therefore, the vegetation cover within the marsh interior is less likely to drive species density than vegetation cover on the marsh edge. To reflect the importance of edge habitat, we measured mangrove cover within 1, 3, and 5 m of the water's edge (Figure 3). Each of the three edge definitions was tested at each of the 16 candidate scales, resulting in 48 combinations of candidate scale and edge definitions. Hereafter, we refer to these permutations of scale and edge definition as habitat definitions. The distribution of seascape variables at each habitat definition is available in Appendix S1: Figures S2–S7.

**FIGURE 3:**
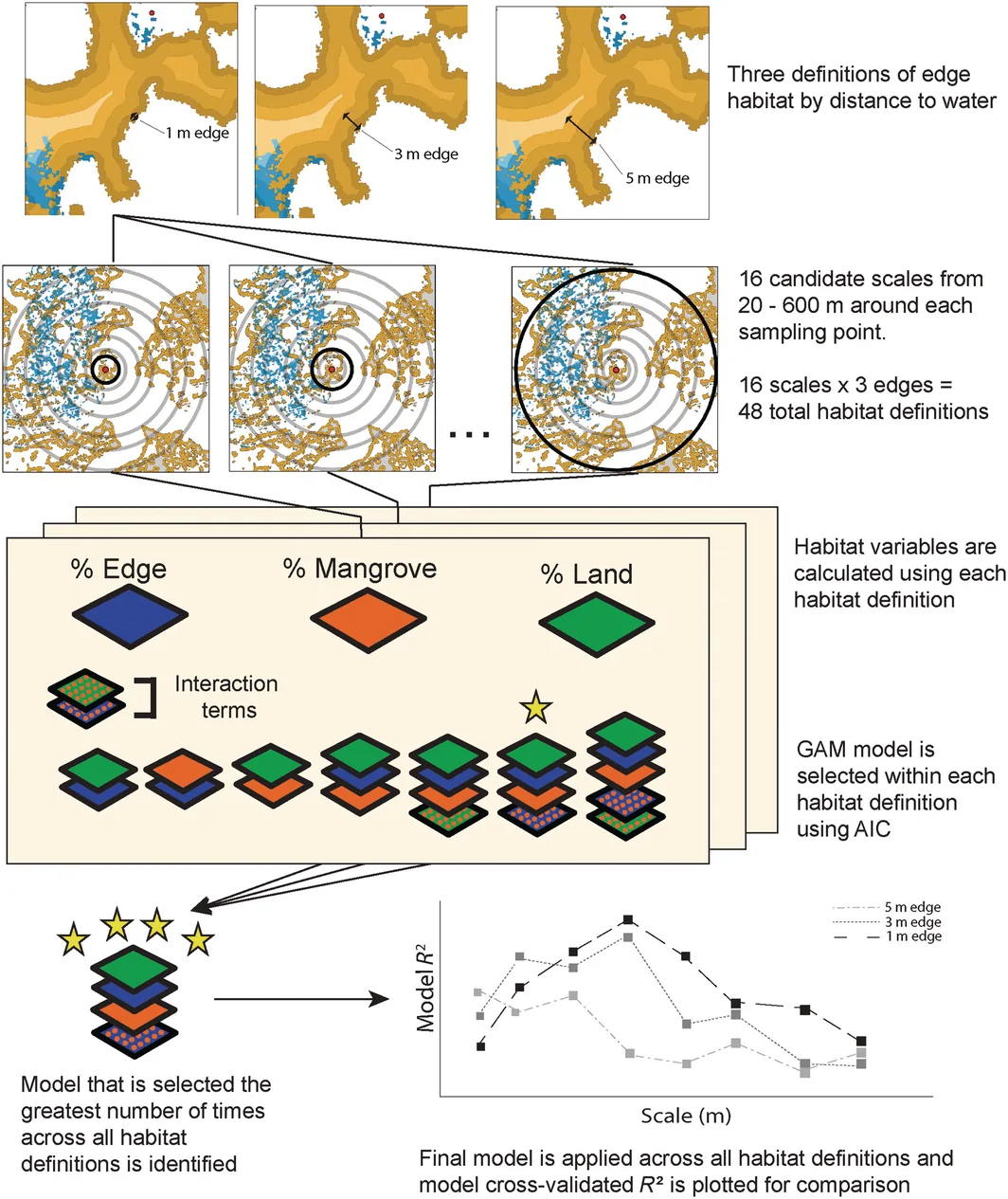
Methodological summary of habitat definition selection process using the drone‐based data. Three habitat‐edge definitions and seven candidate scales were tested. For each permutation of edge and candidate scale, AIC was used to select the best model. The model that was selected most frequently across all permutations was then applied to all habitat definitions.

Within each candidate habitat definition, we calculated percent land cover using the combined area of all land within the candidate scale buffer divided by the area of the candidate scale buffer. We calculated percent edge by determining the percent of the total area of the candidate scale buffer that was defined as edge area using the candidate edge definition. We determined the percent cover of marsh and mangrove vegetation within each habitat scale by measuring the percent of edge area within the buffer that was covered by each vegetation type. Edge area and land/water ratio are commonly used in HSMs in coastal estuaries, but the effect of mangrove cover is not well understood (Smee et al., 2017; Zimmerman et al., 2000). Before the species response analysis, all habitat variables within a candidate habitat definition were standardized with *z*‐scores.

### Selecting scales and extracting partial effects

Our analytical framework uses Akaike information criterion (AIC), smoothing penalties, and k‐fold cross‐validated *R* ^2^ as metrics to select candidate scales of effect and variables of importance at each scale. Multiple scales of effect are frequently observed in multiscale studies (Stuber & Gruber, 2020). Model outputs and variables of importance are visualized across multiple scales (Stuber & Gruber, 2020). While individual models in our workflow were run on single scales, the predictive ability of the model as measured by the k‐fold cross‐validation *R* ^2^ is visualized across multiple scales allowing for multiple scales of effect to be considered for a species in downstream models. For all permutations of 16 candidate scales and 3 candidate edge definitions (48 total candidate habitat definitions), we extracted three landscape variables from the habitat maps as described above: % Land, % Edge, and % Mangrove cover. We also included interaction terms for mangrove cover by percent edge and mangrove cover by percent land. Figure 3 outlines the basic workflow of our scale selection process. Generalized additive models (GAMs) from the R package mgcv (Wood, 2023) were applied within each candidate habitat description to model species abundance at each site. Initially, each GAM model only included variables derived from a single habitat definition/scale. To prevent overfitting, we limited smoothing terms to *k* = 4 knots and interaction terms to *k* = 3 knots. Smoothness selection was performed using maximum likelihood with the double‐penalty approach. The double‐penalty allows the effective degrees of freedom (edf) for uninformative predictors to shrink to near‐0, effectively removing the variable from the model. This is effectively a method of variable selection for GAM models, which often performs better than alternative selection schemes in producing accurate models (Marra & Wood, 2011). We used a cutoff of edf <0.7 to determine whether a variable was meaningfully informative. Since data were continuous, right‐skewed, and could include many zero observations, we used a Tweedie error distribution with a log link which is well suited for biomass and abundance data including zeros without requiring a separate zero‐inflation component (Foster & Bravington, 2013; Shono, 2008). We tested model assumptions using the DHARMa package (Hartig, 2016). Models were tested for dispersion, uniformity, and zero inflation. DHARMa tests zero inflation and dispersion using simulation‐based tests and uniformity is determined using a Kolmogorov–Smirnov of residuals against a uniform distribution (Hartig, 2016). If three or more models for a species failed to meet assumptions (*p* < 0.05 on any of the three tests), then the analysis was re‐run using a negative binomial distribution. Analysis showed that for both drone and satellite data, darter gobies and blue crab models were better suited to a negative binomial distribution, while all other species were run using a Tweedie distribution. If fewer than three models failed, then the failed models were removed from further analysis in the results. In order to simplify GAM models and remove extraneous environmental variables ahead of scale modeling, we compared AIC scores of all possible environmental variable combinations within each candidate habitat description. To facilitate AIC comparisons, models used maximum likelihood estimation rather than restricted maximum likelihood to estimate fit. All models within ±2 ΔAIC of the lowest AIC score were considered candidates. These candidate models were tallied up across all permutations of buffer and edge for a given species. Models from the satellite analysis and the drone analysis were considered separately. The model that was tallied the greatest number of times across all candidate habitat descriptions was then applied in every candidate habitat description. To evaluate the predictive performance of each GAM, we implemented a repeated *k*‐fold cross‐validation procedure. For each species‐scale model, the dataset was partitioned into *k* = 5 folds, or subsets, of approximately equal size. In each iteration, four folds were used for model training and the remaining fold served as the validation set. This process was repeated across all folds and then repeated 10 times with new random fold assignments to reduce variance associated with any single partitioning of the data. In the event that two models were candidates an equal number of times, we selected the model with the highest average cross‐validation *R* ^2^ value across buffers. We selected models using this process for every species using the drone‐based data and the satellite‐based data. The residuals of each permutation's model output were tested for spatial covariance using Moran's I. We also tested every set of variables at each scale for colinearity and removed models with significant colinearity (>0.8). There were three models that met this criterion where % land and % edge were correlated for grass shrimp at 20 and 30 m scales. We plotted cross‐validated *R* ^2^ values from each permutation of species, edge distance, and buffer distance together to identify trends in the explanatory power of the model across a range of buffer distances. The *p*‐values associated with all models are available in the data repositories associated with this study, but we will only apply them in reference to ‘optimized’ models and use a significance threshold of 0.01 to avoid issues of multiple‐testing. For the drone‐based analysis and the satellite‐based analysis, we also calculated how much the cross‐validated *R* ^2^ changed, on average, when the measurement scale was altered by 10 m. This metric gives an indication of how sensitive the models are to spatial scale.

To identify changes in the partial effect of singular habitat variables over scales, we plotted partial effects for each significant variable in the model across all permutations. We generated partial effect plots by incrementally scaling the variable of interest while keeping all other variables at their mean and plotting the GAM model's predicted values. We kept habitat variables standardized across scales to better model species response over gradients present within each scale.

### Habitat suitability maps

To compare and assess suitability models, we projected a subset of models onto our study domain to produce raster maps of predicted species abundance. We used the R package *terra* (Hijmans, 2020) to calculate habitat metrics for each pixel within a subregion of our study site. Satellite‐based models were projected onto a region encompassing 15 sampling sites, and drone‐based models were projected onto a subset of that region encompassing seven sampling sites. When drone and satellite‐trained models were compared, drone‐trained models were projected onto drone‐derived habitat rasters and satellite‐trained models were projected onto satellite‐derived habitat rasters. Model predictions were compared by plotting the difference in predicted species abundance for each pixel on the seascape. Sample sites were color‐coded by the model with the lowest absolute difference between prediction and the true value at that site. We also calculated the average mean out‐of‐sample prediction for each site during each iteration of the cross‐validation protocol. This gives us an estimate of the model's ability to predict each site if it were not included in the training data. Comparing out‐of‐sample predictions among models and across sites helps reveal whether models capture different subsets of sites more effectively, or whether they perform similarly on the same locations. To see whether powerful models behave similarly across scales or diverge and explain different relationships between species and seascape, suitability models were compared between different scales with similar predictive power. We also compared high and low‐power models to quantify possible consequences of misjudging scale of effect. Finally, drone‐trained and satellite‐trained models were compared to better visualize how each data type informs models on identical seascapes.

## RESULTS

### Explanatory power

Landscape variables used in this analysis predicted species abundance moderately well for blue crabs and white shrimp, but not for grass shrimp and darter gobies. For the drone‐based analysis (20–150 m habitat radius) the highest cross‐validated *R* ^2^ was 0.337 for white shrimp and 0.223 for blue crab (Figures 4 and 5). For the larger scale satellite‐based analysis (70–600 m habitat radius), the highest cross‐validated *R* ^2^ value was 0.220 for blue crab and 0.314 for white shrimp (Table 1).

**FIGURE 4:**
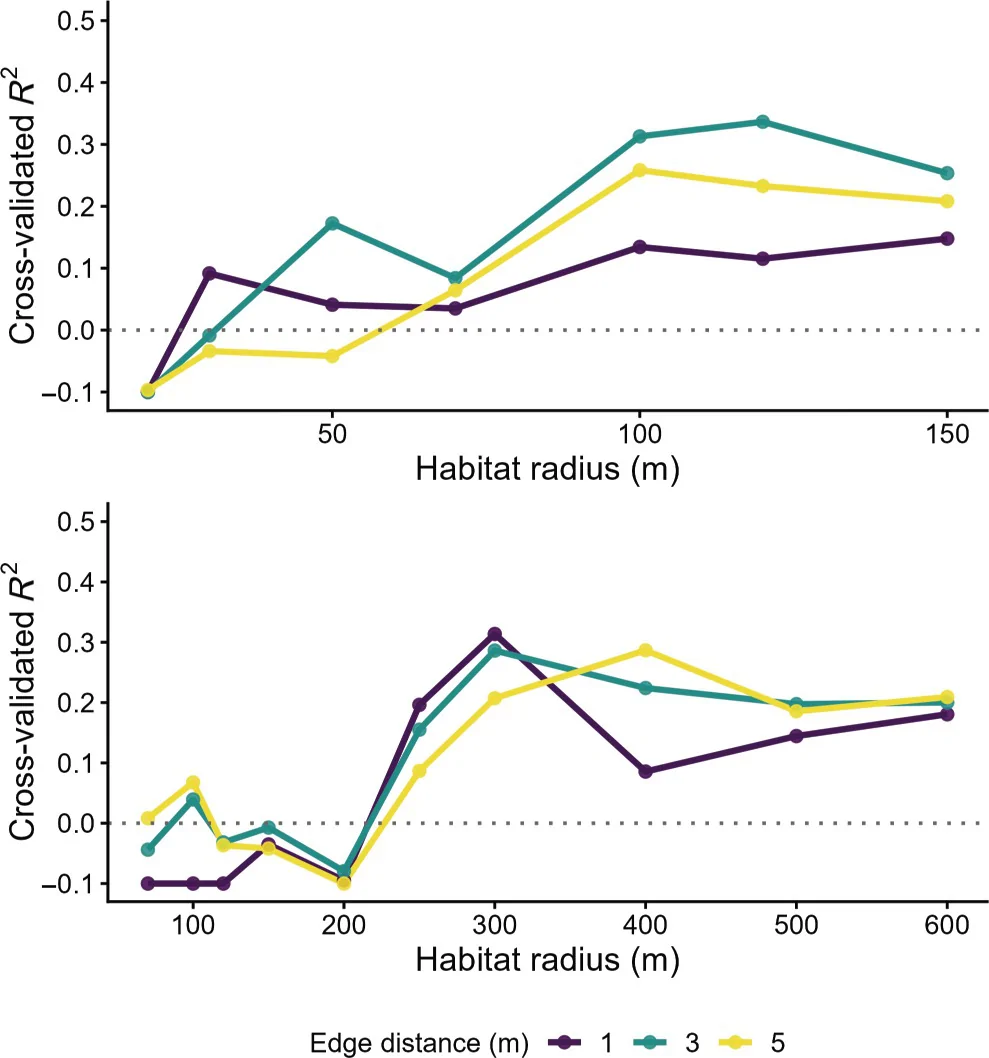
Plotted GAM model outputs for white shrimp using drone (top) and satellite‐based (bottom) data. Each plot shows mean *R* ^2^ value of the model on out‐of‐sample cross‐validation. Models with a cross‐validated *R* ^2^ < −0.1 were set to −0.1 for plotting.

**FIGURE 5:**
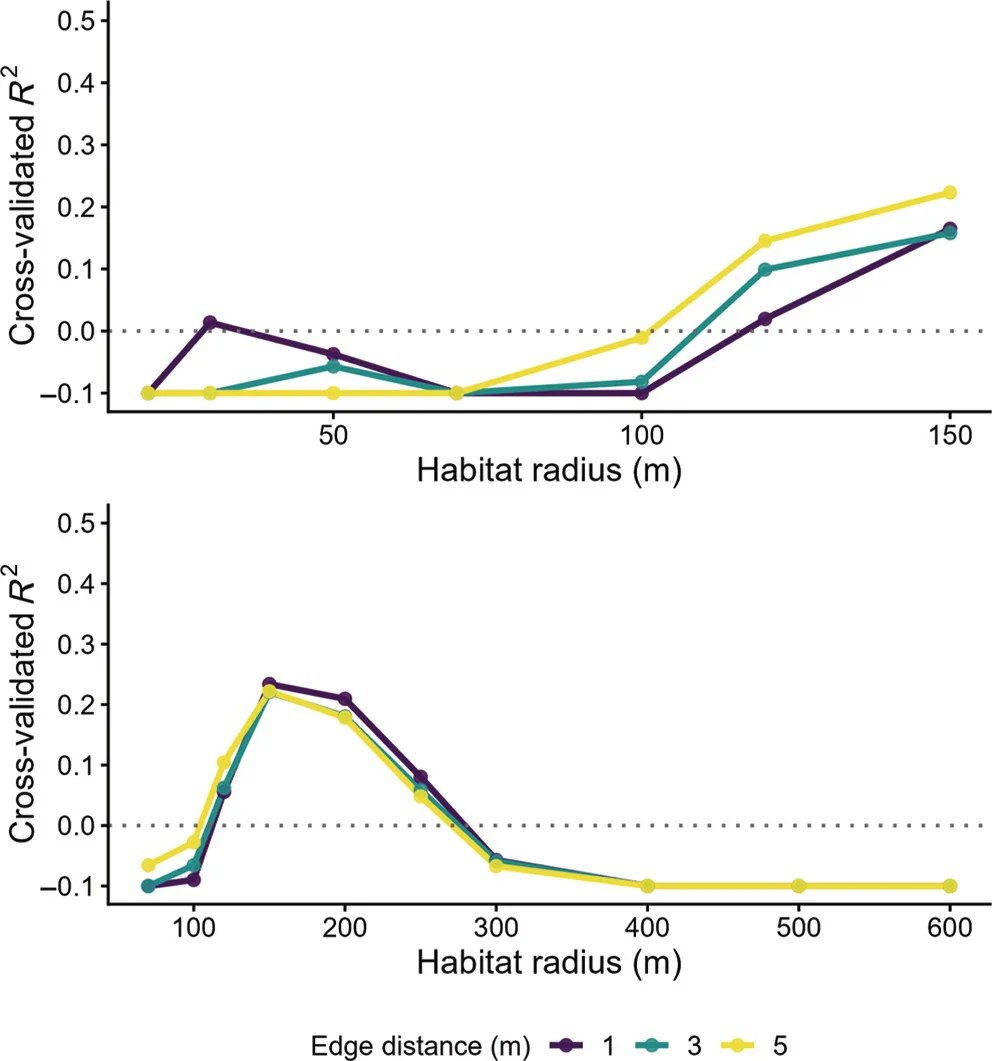
Plotted GAM model outputs for blue crab using drone (top) and satellite‐based (bottom) data. Each plot shows mean *R* ^2^ value of the model on out‐of‐sample cross‐validation. Models with a cross‐validated *R* ^2^ < −0.1 were set to −0.1 for plotting.

**TABLE 1:** Habitat variables included in the final model for each species at each scale.

Species	Data	Cross‐validated *R* ^2^	Optimal definition (meters)		% Edge	% Land	% Mangrove	Mangrove‐land interaction	Mangrove‐edge interaction
			Buffer	Edge					
Blue Crab	Satellite data	0.220	150	1	~0	1.8	~0	~0	~0
	Drone data	0.223	150	5	~0	1.7	~0	~0	NA
White Shrimp	Satellite data	0.314	300	1	0.95	~0	1.4	~0	0.86
		0.287	400	5	0.95	~0	0.75	~0	0.79
	Drone data	0.172	50	3	0.79	~0	0.89	~0	0.88
		0.337	120	3	0.83	1.74	0.87	~0	0.90

*Note*: Values beneath each variable represent the edf value of the given variable in the model at the optimized scale (highest cross‐validated *R* ^2^). Values approaching zero indicate the variable was essentially excluded from the model. NA values indicate the variable was excluded during the AIC checking stage of the workflow and was not considered in the optimized model. Analyses that indicated no predictive models (grass shrimp and darter gobies) are not included in the table.

Scale also had an effect on model performance for blue crabs and white shrimp. Analyses using drone and satellite data were roughly equal in their sensitivity to scale for blue crabs, with the model's predictive power changing by an average of 1.4 percentage points for every 10‐m change in buffer size. For white shrimp, the drone‐based analysis was more sensitive to scale, with an average change of 2.9 percentage points for every 10‐m change in buffer size. For blue crabs and white shrimp, the cross‐validated *R* ^2^ values ranged from below 0 on some scales to 0.223 and 0.337 at the maximum, respectively.

### Variable and scale selection

Apparent scale of effect as estimated by peaks in the cross‐validated *R* ^2^ of the model was clearly indicated in two of the four species, with species‐specific peaks occurring within both the drone‐based and satellite‐based scale ranges. White shrimp and blue crabs showed well defined peaks in predictive power of habitat models at key scales. For blue crabs, this peak was unimodal, but multiple peaks in model predictive power are indicated for white shrimp. Grass shrimp and darter gobies showed no clear peaks in the data, and models generally performed poorly in predicting out‐of‐sample data (Appendix S1: Figures S8 and S9). For both blue crab and white shrimp, the AIC‐based model selection process selected models with each variable (% Land, % Edge, and % Mangrove) as well as the interaction of % Mangrove and % Edge. The interaction between % Mangrove and % Land was selected for all but the drone‐based blue crab model. However, not all variables were informative in the optimized models. A threshold of edf >0.7 and a *p* < 0.01 was used to determine variable importance to ensure the predictor improves fit (edf) and has a significant effect (*p*‐value). For blue crab, % Land was the only informative variable at the optimized scale (edf >0.7 and *p* < 0.01) in both the drone‐based and satellite‐based analysis. White shrimp had two ‘peaks’ in model performance for both the drone and satellite‐based analysis. These peaks were at 300 and 400 m for satellite‐based analysis, and at 50 and 120 m for drone‐based analysis. % Edge was considered informative for all these scales. % Mangrove was important for all but the 400‐m model, and % Land was only informative for the 120‐m model. For white shrimp, the interaction between % Mangrove and % Edge was also informative at all predictive scales. The partial effect of this interaction term indicated that the relationship between abundance and % Mangrove cover was much stronger when % Edge was also high. At higher % Edge values, white shrimp were more abundant at intermediate‐high mangrove cover sites. When % Edge was low, there was not a strong relationship between % Mangrove cover and abundance.

### Multiscale models

Multiple scales of effect were apparent for the white shrimp data, indicated by distinct peaks in cross‐validated *R* ^2^ at different scales. This could indicate multiple drivers of species density operating on different scales (Figure 4). To determine the drivers of these peaks, we included habitat variables from all peak scales (Table 1) into a GAM model to test whether predictive power improved when considering metrics from multiple different scales. In the full model, % Mangrove at 50 m, % Land at 120 m, % Edge at 400 m, and the interaction of % Mangrove and % Edge at 120 m were informative variables (edf >0.7, *p* < 0.01). When these variables were included in their own model, cross‐validated *R* ^2^ was high at 0.30, but lower than the best single‐scale models at 300 and 120 m alone (Table 1). This process was also repeated with just the data from 300 and 120 m, the two best performing scale definitions. In this model, % Mangrove at 120 m, % Edge at 300 m, and the interaction of mangrove and edge at 120 m were informative. The cross‐validated *R* ^2^ for the final model was 0.25, somewhat lower than either scale on its own.

### Predictive maps

We compared three sets of models by projecting predictions onto seascape rasters. Our first set was a comparison of two high‐performing candidate habitat definitions for white shrimp: buffer = 300 m, edge = 1 m and buffer = 400 m, edge = 5 m. Both models included % Edge and % Mangrove as the driving variables (Figure 6). Partial effects of these variables indicate a slightly greater predicted magnitude associated with high % Edge. % Mangrove had a smaller partial effect but showed increasing abundance up to intermediate levels of mangrove cover with a slight decrease at very high levels of mangrove cover. Disagreement in the two models showed that the 400‐m model predicted greater abundances in regions surrounded by land, where the 300‐m model typically predicted higher abundances in more open water. For the 15 sites in the region, the 300‐m model was a better fit for 5 of them. Out of sample predictions were similar across all sites, suggesting the two models performed very similarly overall, and are likely explaining the same variability in species abundance.

**FIGURE 6:**
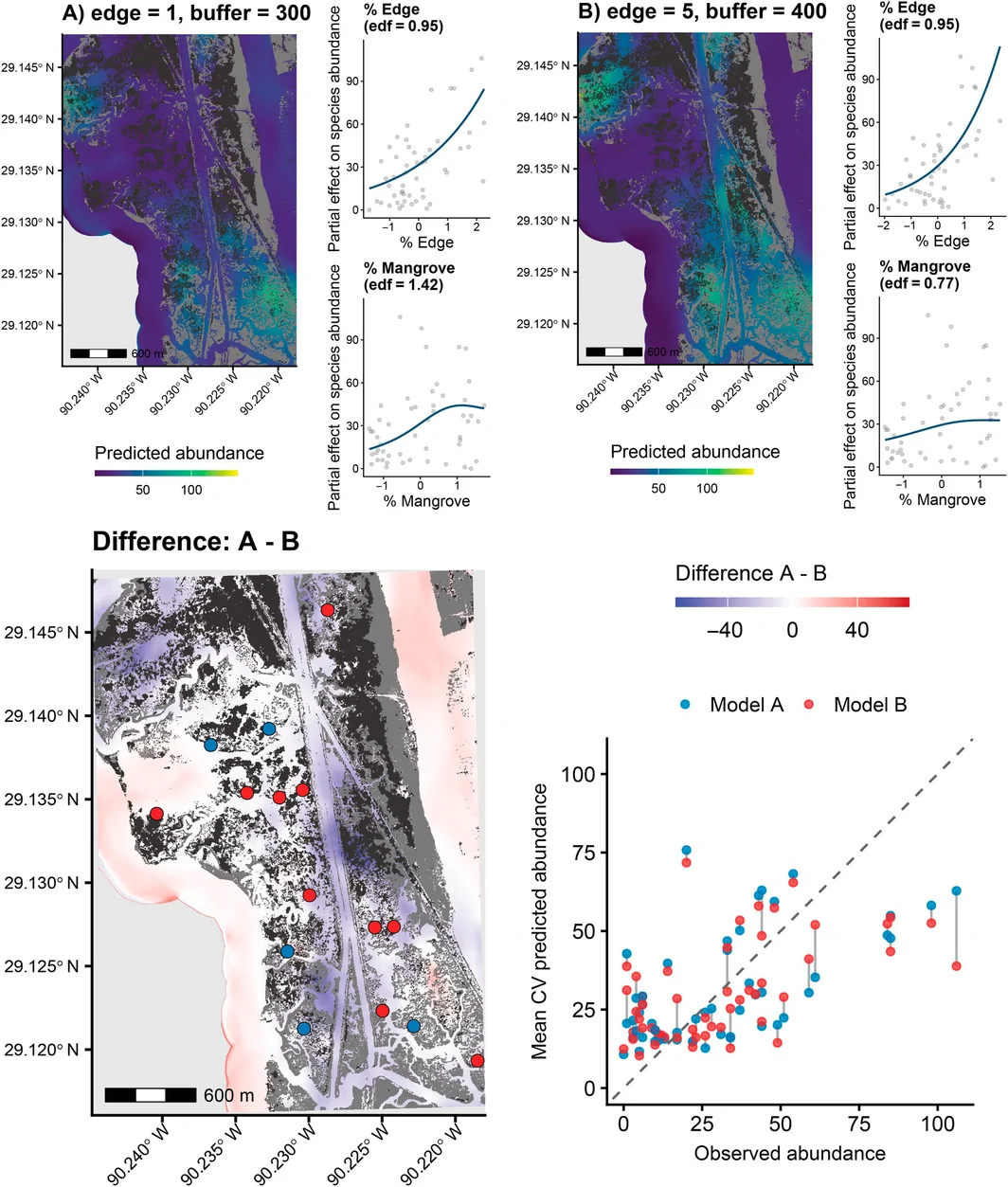
Comparison of model predictions from different scales. Map A shows the predicted abundance of white shrimp across a region of the study area based on the habitat model trained on seascape metrics within 300 m of the sample site and using 1 m as a definition for edge habitat. Map B shows the predicted abundance of white shrimp in the same region based on the habitat model trained on seascape metrics within 400 m of the sample site and using 5 m as a definition for edge habitat. The predictive power of model A (cross‐validated *R* ^2^ = 0.31) was slightly higher than the predictive power of model B (cross‐validated *R* ^2^ = 0.29) during cross‐validation. The partial effects of the variables of importance in each model are shown to the right of their respective prediction map. The bottom left figure shows the prediction disagreements between the two models calculated as the predicted abundance of model A—predicted abundance of model B. Points on the bottom left figure represent sampling sites colored according to whether predicted values of model A (blue) or model B (red) were closer to observed values. The scatterplot on the bottom right shows observed abundance versus Mean out‐of‐sample predictions for both models at every site. Lines are drawn between points representing the same site to highlight differences in model predictions.

The second set of maps compared the highest and lowest performing candidate habitat definitions for white shrimp. These were buffer = 300 m, edge = 1 m and buffer = 200 m, edge = 3 m (Figure 7). Once again, % Edge and % Mangrove were the only informative variables in both models. Partial effects of these variables show a smaller difference in partial effect of % Mangrove over the range of values in the 200‐m model compared with the 300‐m model. This indicates that mangrove cover has a weaker effect on final predictions in the 200‐m model. The out‐of‐sample estimates for model B showed several sites with very high out of sample predictions but low observed abundance.

**FIGURE 7:**
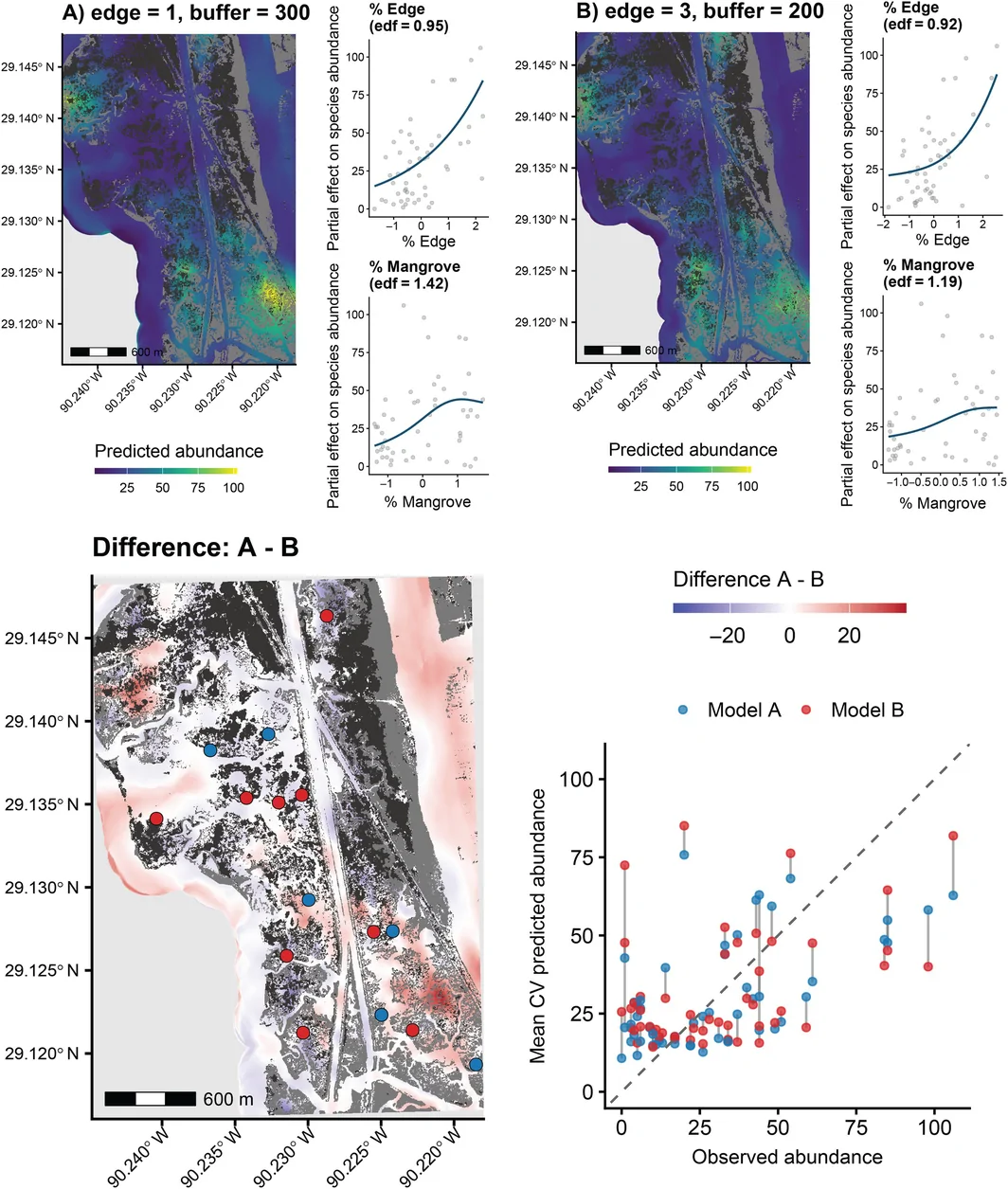
Comparison of model predictions from different scales. Map A shows the predicted abundance of white shrimp across a region of the study area based on the habitat model trained on seascape metrics within 300 m of the sample site and using 1 m as a definition for edge habitat. Map B shows the predicted abundance of white shrimp in the same region based on the habitat model trained on seascape metrics within 200 m of the sample site and using 3 m as a definition for edge habitat. The predictive power of model A (cross‐validated *R* ^2^ = 0.31) was shown to be substantially higher than the predictive power of model B (cross‐validated *R* ^2^ < 0) during cross‐validation. The partial effects of the variables of importance in each model are shown to the right of their respective prediction map. The bottom left figure shows the prediction disagreements between the two models calculated as the predicted abundance of model A—predicted abundance of model B. Points on the bottom left figure represent sampling sites colored according to whether predicted values of model A (blue) or model B (red) were closer to observed values. The scatterplot on the bottom right shows observed abundance versus mean out‐of‐sample predictions for both models at every site. Lines are drawn between points representing the same site to highlight differences in model predictions.

Blue crabs did not show the same pattern of multiple highly predictive habitat definitions as white shrimp, but there was a clear commonality between drone‐ and satellite‐based data, with both indicating 150 m as a highly predictive scale. We made comparisons between the drone‐based and satellite‐based model at buffer = 150 m, edge = 5 m to understand how differences in resolution and landscape measurement tools influenced model outputs (Figure 8). Both models showed a similar unimodal relationship with % Land. The satellite‐trained model predicted the same range of abundances (0–4) over the comparison area as the the drone‐trained model (0–4). Models generally showed agreement across the comparison area with localized areas showing stronger divergence. Despite this, models compared very similarly in terms of their out‐of‐sample predictions and their overall cross validation scores. Observed versus predicted scatterplots and partial effect plots show that both models could not predict the presence of high‐abundance sites and sometimes overestimated abundance at high % Land sites. While out‐of‐sample site predictions were similar, the use of satellite versus drone data in this instance would generate locally divergent predictions of blue crab abundance in the study area.

**FIGURE 8:**
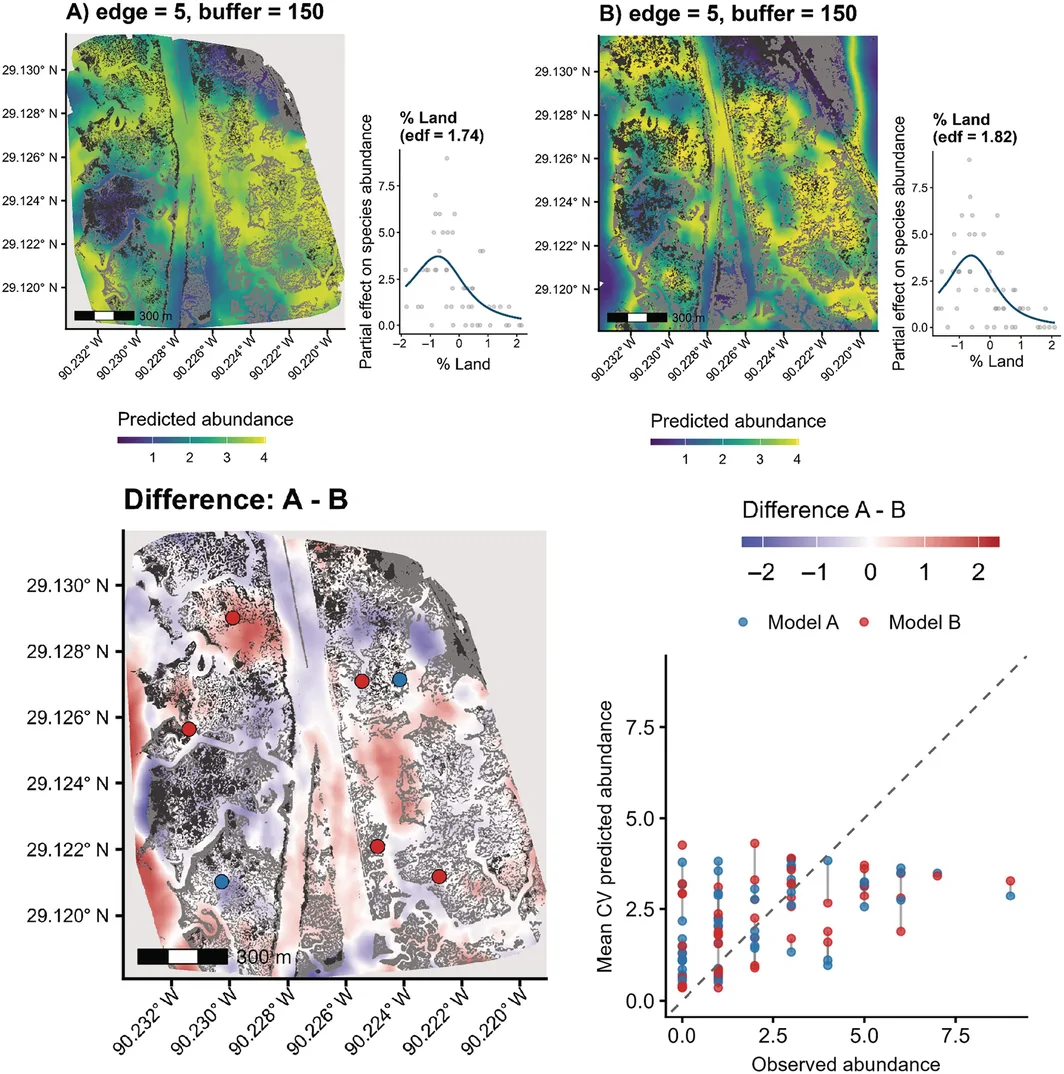
Comparison of model predictions from different scales. Map A shows the predicted abundance of blue crab across a region of the study area based on the habitat model trained on drone‐derived seascape metrics within 150 m of the sample site and using 5 m as a definition for edge habitat. Map B shows the predicted abundance of blue crab in the same region and using the same metrics, except the model uses satellite‐derived seascape metrics. Mangroves are shown in gray, and marsh grass is shown in black. The predictive power of the drone‐based model A (cross‐validated *R* ^2^ = 0.22) was identical to the predictive power of the satellite‐based model B (cross‐validated *R* ^2^ = 0.22) during cross‐validation. The partial effects of the variables of importance in each model are shown to the right of their respective prediction map. Sites within the region are shown as points colored according to the model that most closely predicts their abundance. The map on the lower left shows the prediction disagreements between the two models calculated as the predicted abundance of model A–predicted abundance of model B. Points on the bottom left figure represent sampling sites colored according to whether predicted values of model A (blue) or model B (red) were closer to observed values. The scatterplot on the bottom right shows observed abundance versus mean out‐of‐sample predictions for both models at every site. Lines are drawn between points representing the same site to highlight differences in model predictions.

In all comparisons, the overall relationship between abundance and seascape structure remained fairly consistent. For example, across all predictive scales, white shrimp had a positive relationship with % Edge habitat, and blue crabs had a unimodal relationship with % Land. For white shrimp, % Mangrove cover had a more variable relationship with scale, with some smaller scales showing mostly linear positive relationships, and some larger scales showing more unimodal relationships with a maximum partial effect at mangrove‐heavy mixed sites (*z*‐score ~0.5). Figures showing partial effects across all variables and scales are available in supplementary materials (Appendix S1: Figures S10 and S11). Although gross relationships between abundance and habitat metrics were sometimes preserved across scales, differences between scales were enough to meaningfully change predictions and predictive power. Habitat suitability map comparisons showed that models at different scales with similar variables describing similar overall abundance‐habitat relationships can predict divergent patterns across a landscape. Although the habitat maps differed among models, their prediction errors were highly consistent: Models tended to struggle on the same sites and performed well on the same sites, even when their spatial prediction patterns diverged.

## DISCUSSION

Our approach generated HSMs for white shrimp and blue crab, but models for darter goby and grass shrimp had extremely low predictive power possibly indicating missing variables or scales of effect outside the range of the study. For white shrimp and blue crab, scale made a meaningful difference in the predictive power of the model in k‐fold cross validation tests. These species showed different responses to landscape structure with different variables driving abundance. White shrimp had an unexpected positive relationship with the climate migrant foundation species *Avicennia germinans*, and the amount of edge habitat and land cover were informative for white shrimp and blue crabs respectively. This indicates a meaningful relationship between abundance and landscape structure that is highly dependent on species and the scale of measurement. The landscape metrics used in this study are particularly sensitive to the effects of marsh erosion (Jerabek et al., 2017), sea level rise (Phillips, 2018), and warming climates (Osland et al., 2017), suggesting a strong possibility for changes in species abundance as climate change intensifies.

### Scale selection and model performance

White shrimp and blue crab both show a clear rise and fall of predictive power over the range of scales in our analysis, which suggests that the true scale of effect was within our range of scales considered (Jackson & Fahrig, 2015). Grass shrimp showed spatial structure in their distribution across the habitat, as apparent by significant values of Moran's I, but cross‐validation revealed that there was very little out of sample predictive power in habitat models. A major challenge in habitat suitability modeling is accounting for spatial autocorrelation, which can occur when species have a patchy distribution that is not explained by environmental drivers in the model. Spatial autocorrelation can inflate Type I error rates if it is not considered (Diniz‐Filho et al., 2003). We found that some models at smaller scales exhibited significant spatial covariance in residuals, likely due to their failure to capture habitat characteristics at larger, more relevant extents. For grass shrimp, all scales had significant spatial covariance, but models had low predictive power. This suggests that for grass shrimp abundance the drivers of habitat use were not captured in these models. The discrepancies in predictive power between species is likely rooted in their biology and behavior. Grass shrimp and darter gobies have less explicit estimates of home range in the literature, but grass shrimp are known to have very high site fidelity (Allen et al., 2015; Wessell et al., 2001), and gobies tend to have comparatively small home ranges (Ray & Corkum, 2001). These characteristics could make population‐level HSMs more challenging, since these species are likely selecting for microhabitat access rather than seascape variables on the scale of 10s to 100s of meters.

For the other two species in our analysis, apparent scales of effect and seascape relationships were largely in agreement with established literature. White shrimp are known to have a strong relationship with marsh edge habitat and have approximate home radii of 200–300 m (Minello & Rozas, 2002; Webb & Kneib, 2002, 2004). Our analysis identified 300 m as one of the best scales for modeling species‐habitat relationships and found a strong relationship between percent edge habitat and white shrimp abundance (*p* < 0.001, cross‐validated *R* ^2^ = 0.31) (Figure 4). Blue crab densities are also related to the availability of edge habitat and land‐to‐water ratios, with ideal ranges between 40% and 80% land cover (Minello & Rozas, 2002). Our method identified a similar relationship between blue crab density and percent land cover within 150 m of the sampling site (Figure 5). This scale of effect aligns with the average home range of juvenile blue crabs on Georgia's Atlantic coast (Wrona, 2004).

For blue crabs and white shrimp alike, out of sample predictive power was highly variable across relatively small changes in scale, going from zero to its maximum value with changes in scale of only 100 m (Figures 4 and 5). This variability supports evidence that HSMs' performance can be sensitive to relatively small changes in measurement scale. In systems where assumptions about species landscape use are incorrect, imprecise, or outdated because of disturbance (Cannizzo & Griffen, 2016; Mayer et al., 2019), HSMs could lose some of their predictive power.

### Habitat relationships across scales

There is general consensus that multiscale models, or models that include variables from multiple scales, can enhance the accuracy of predictions since the variables which inform habitat selection change as the scale of observation changes (McGarigal et al., 2016; Rodemann et al., 2025; Sánchez et al., 2014; Stuber & Fontaine, 2019; Zeller et al., 2014). For white shrimp, the only species with multiple potential scales of effect, the informative variables at the two descriptive scales were the same, and the partial effects of those variables were similar. The cross‐validation accuracy of multiscale white shrimp models was not better than the single‐scale models. In light of these findings, it is likely that the distinct peaks observed are describing the same fundamental relationship between white shrimp abundance and landscape rather than picking up on multiple processes occurring on different scales. While multiscale models are typically more descriptive and accurate, there is precedent for studies finding little (Boscolo & Metzger, 2009; Graf et al., 2005) or no (Martin & Fahrig, 2012) improvement in multiscale models over single‐scale models. Habitat generalists may be less likely to have strong responses at multiple scales, since their habitat requirements are less exacting than specialists (Martin & Fahrig, 2012). This study also includes relatively small scales and may not span more than one level of organization. There is greater evidence for distinct landscape relationships occurring on multiple hierarchical levels of organization rather than adjacent spatial scales (Stuber & Fontaine, 2019).

### Application for restoration and conservation ecology

The ability to identify scale‐dependent habitat relationships has direct applications in habitat restoration and conservation planning. Restoration efforts that fail to consider the appropriate spatial scales that species interact with their environment may create suboptimal habitat conditions for target species and compromise conservation goals (Rozas et al., 2005). Our results suggest that predictive power of abundance models can change substantially over small changes in chosen scale of effect. We also find that relatively minor differences in partial effects of each variable in our HSMs can drive divergent predictions across the marsh landscape.

Our results reinforce the important role of seascape configuration for estuarine species. Other studies have established that white shrimp and blue crabs are tightly linked to metrics like edge habitat and measures of marsh fragmentation (Boström et al., 2011; Haas et al., 2004; Minello & Rozas, 2002); however, the effect of mangrove encroachment on species abundance is not well known (Osland et al., 2022). Mangrove encroachment presents a stark change in habitat structure and potentially habitat access for estuarine species (Caudill, 2005; Yando et al., 2016). Differences in the quality of *Spartina* and mangrove detritus also raise the possibility of altered energetic pathways for estuarine consumers (Armitage et al., 2021; Goeke et al., 2024; Nelson, Lesser, et al., 2019). Comparative studies of marsh and mangrove habitats have generated variable results. Smee et al. (2017) found that white shrimp and blue crabs were less abundant in marshes surrounded by mangroves, while a study by Caudill (2005) showed that the abundance of white shrimp is higher in mangrove habitats. We found an overall positive relationship between white shrimp and mangrove cover, with intermediate levels of mangrove cover having greater abundance at the larger scales. Interestingly, models also indicated an important interaction between % Edge and % Mangrove on white shrimp. % Mangrove only increases predicted abundance when % Edge is also higher. When edge habitat is low, mangrove edge cover has little effect abundance. The % Edge variable initially defines the amount of habitat available to consumers, and % Mangrove defines how much of that habitat is mangrove‐dominated. Taken together, our results suggest that mangroves may enhance habitat quality for white shrimp. However, this study does not indicate a clear mechanism driving this pattern. Possible changes in predation rates of white shrimp in dense mangrove stands (Scheffel et al., 2017), or more efficient feeding at higher trophic positions (Nelson, Lesser, et al., 2019), may improve survivorship. W*i*thout a clear mechanism linking mangrove habitat to increased support for white shrimp, it remains difficult to predict how this pattern will develop as mangrove encroachment progresses.

Combining drone and satellite imagery allowed us to model habitat suitability at scales that would not have been possible using either tool alone. Drone‐based and satellite‐based models had almost identical predictive performance for blue crabs when compared on the same scale, although the projected abundance predictions diverged in localized areas of the seascape. While satellite data performed well for models at larger scales, it is ultimately limited in its ability to detect relationships at small scales. For example, the highly predictive relationships at 120 m for white shrimp are only evident in the drone data. Satellite data showed systematic bias in underestimating % Edge and % Mangrove metrics when compared with drone data, and bias became more extreme as spatial scale decreased (Appendix S1: Figure S1). Satellite data can have a wide range of spatial resolutions from <1 m in limited areas generated by commercial microsatellites (Sandau, 2004) to tens of meters in global datasets available publicly and with a wide range of spectral bands (Stewart et al., 2023). Low‐resolution imagery is insufficient for testing small‐scale (<100 m) species‐habitat relationships, but high‐resolution satellite and drone data are also limited by the spatial extent that can be realistically characterized. Even using a fixed‐wing drone designed for extensive surveys, we could still only capture an area ~150 m around all of our sampling sites. The decision between using satellite versus drone‐based imagery in similar models should be contingent on the anticipated scale of effect for the process in question. In many cases, using both of these data sources together will allow for a more comprehensive evaluation of spatial scales where species‐landscape and ‐seascape relationships may be occurring and scale of effect is unknown.

### Future directions and applications

Our approach is broadly applicable beyond the species and habitats examined in this study. Scale‐optimization methods can be extended to assess the role of scale in a variety of spatially explicit ecological processes, including species distributions, population dynamics, and metrics of ecosystem function. Seascape configuration can have meaningful effects on trophic niche and the availability of energetic resources (James et al., 2021; Santos et al., 2022), and the explanatory power of these relationships could be enhanced by empirically identifying the spatial scale that maximizes explanatory power. Future studies could explore the use of machine learning approaches, such as random forests or ensemble modeling, offering robust cross‐validation and improved predictive performance over traditional statistical models. Machine learning models are useful for managing many variables and complex interactions, but producing simple generalizable relationships is often challenging. Given the accelerating pace of environmental change, particularly in coastal systems, it is essential to ensure that HSMs remain flexible and responsive to shifting habitat dynamics. By systematically assessing habitat relationships across scales, we can improve the accuracy and applicability of these models for conservation planning, ultimately supporting more effective conservation and management strategies in the face of global change.

## CONFLICT OF INTEREST STATEMENT

The authors declare no conflicts of interest.

## Supporting information


Appendix S1.

## Data Availability

Data and code are available in the Biological and Chemical Oceanography Data Management Office (BCO‐DMO) as follows: species occurrence records from Leavitt et al. (2025e) at https://doi.org/10.26008/1912/BCO-DMO.947784.1 (see the file named PtFouSept2022count_ns.csv); shapefiles with classified habitat based on (i) drone imagery from Leavitt et al. (2025a) at https://doi.org/10.26008/1912/BCO-DMO.947918.1 and (ii) satellite imagery from Leavitt et al. (2025b) at https://doi.org/10.26008/1912/BCO-DMO.947958.1; code for calculating habitat variables at the spatial scales and output data from Leavitt et al. (2025d) at https://doi.org/10.26008/1912/BCO-DMO.947975.1 and Leavitt et al. (2025c) at https://doi.org/10.26008/1912/bco-dmo.948112.1, for satellite and drone data, respectively; code for habitat suitability model comparison and selection, as well as model outputs and summaries, from Leavitt et al. (2026) at https://doi.org/10.26008/1912/bco-dmo.948167.2.
