# Supplementary material for: Importance of scale in seascape habitat suitability modeling: Predicting species distributions in changing ecosystems
Source: Ecol Appl. 2026 Nov 4;36(7):e70318. doi: 10.1002/eap.70318 (PMC13735485; doi:10.1002/eap.70318)
Supplement: Supplementary file 1 [file EAP-36-e70318-s001.pdf]

Appendix S1

Importance of scale in seascape habitat suitability modeling: Predicting species distributions in changing ecosystems

Herbert Leavitt, Alexander Thomas, James A. Nelson

Ecological Applications

Table S1. Comparison of drone- and satellite-derived habitat classifications using class-specific accuracy metrics: User's Accuracy (precision), Producer's Accuracy (recall), F1 score, and IoU.

Habitat Class	User Accuracy	Producer Accuracy	F1	IoU
Water	0.776	0.779	0.778	0.636
Mangrove	0.718	0.637	0.675	0.51
Spartina	0.521	0.636	0.573	0.402
Artificial	0.6	0.333	0.429	0.273

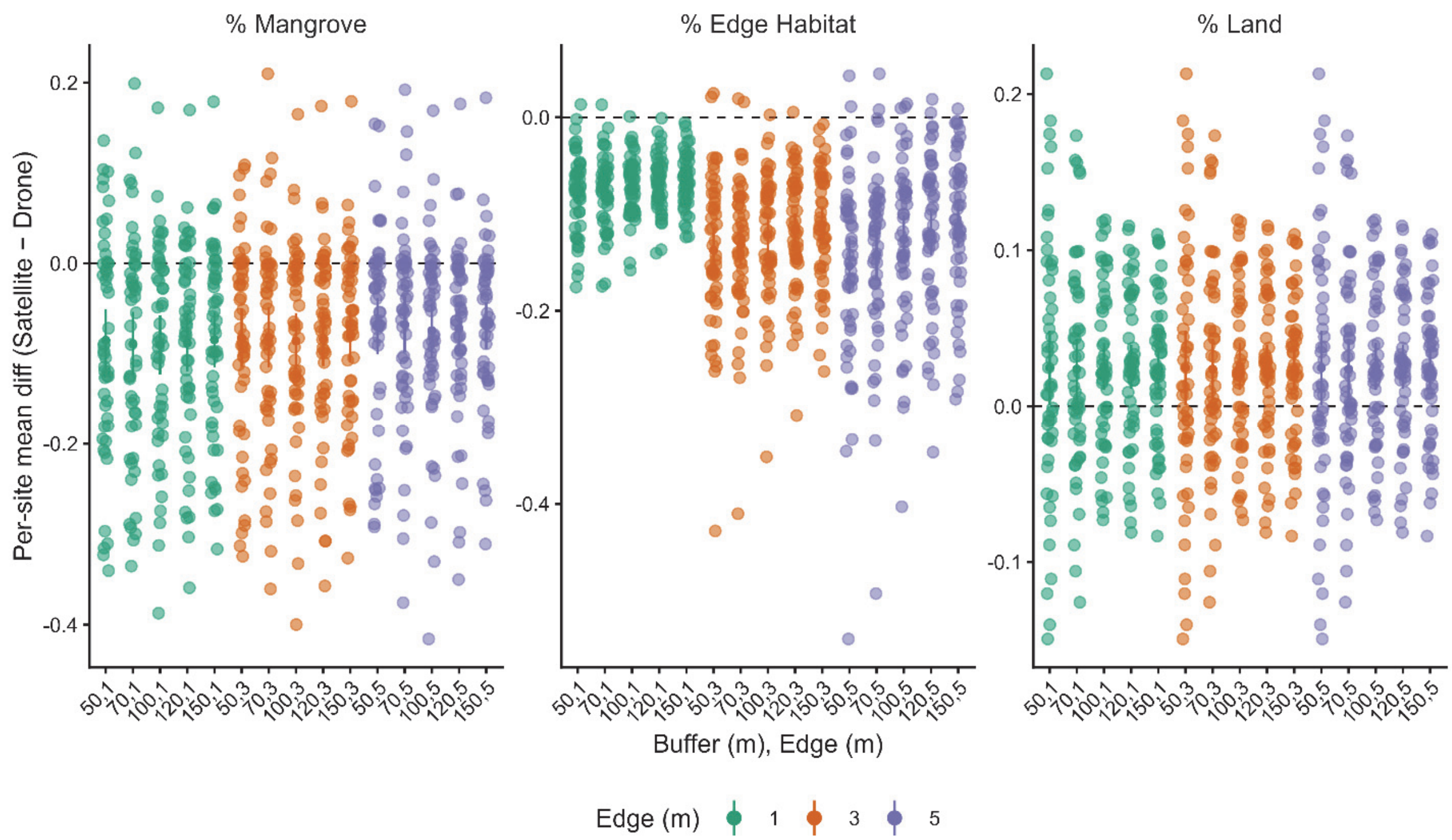

Figure S1 The mean difference between landscape metric values calculated using drone-based imagery and satellite-based imagery at identical spatial scales. Difference is calculated as satellite-derived value – Drone derived value. Drone imagery has a resolution is 8.37 cm/pixel, and satellite imagery is 2.8 x 3.3 meters per pixel.

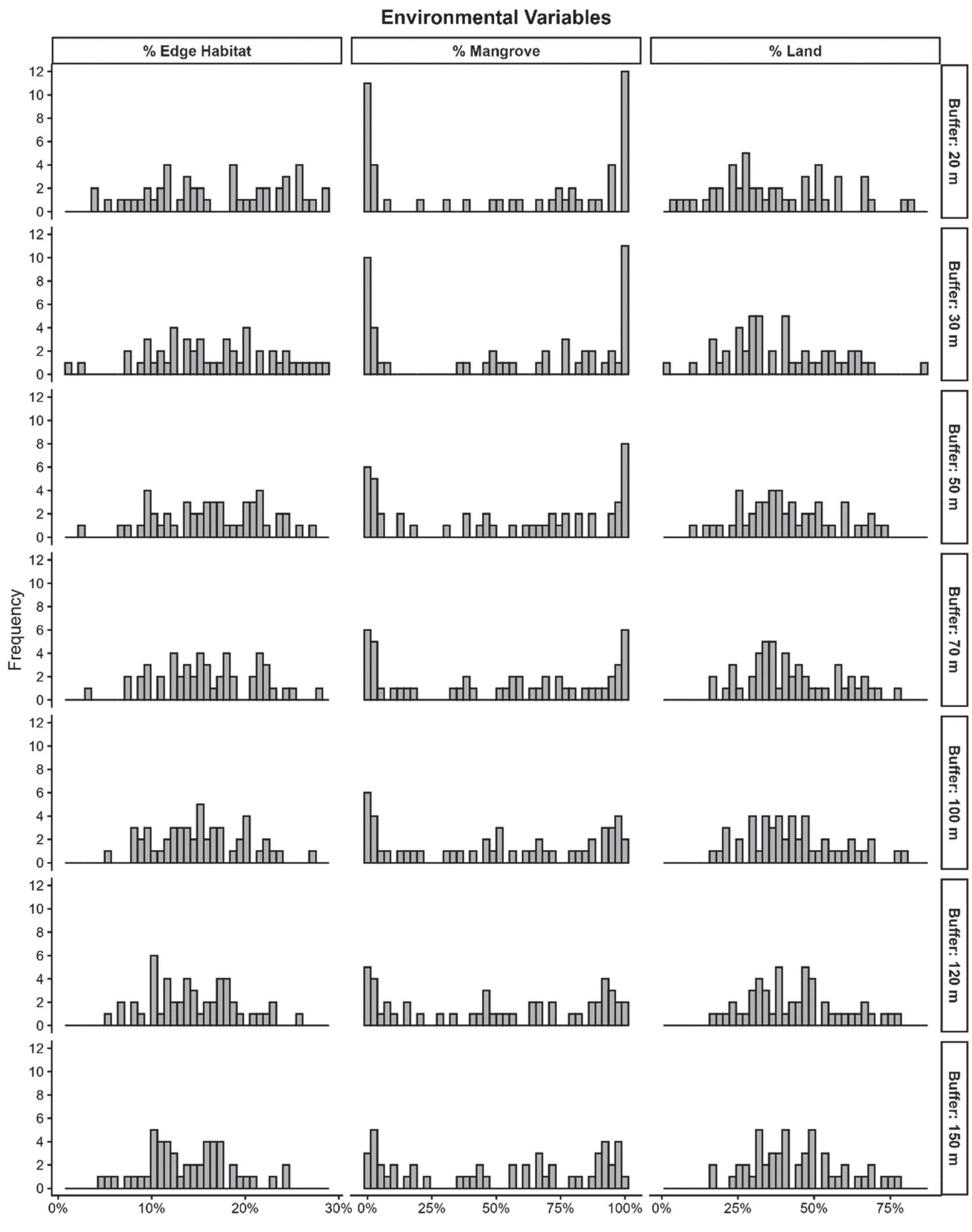

Figure S2. Histograms of habitat variables from all 52 sampling sites calculated at each candidate scale of effect using drone-based habitat imagery. Sites were chosen based on spatially stratified random sampling to optimize for mangrove cover at 200 meters. Columns are the three habitat variables we used, and each row is a candidate scale used in the modeling workflow. Metrics were calculated with edge defined as land within one meter of the water's edge.

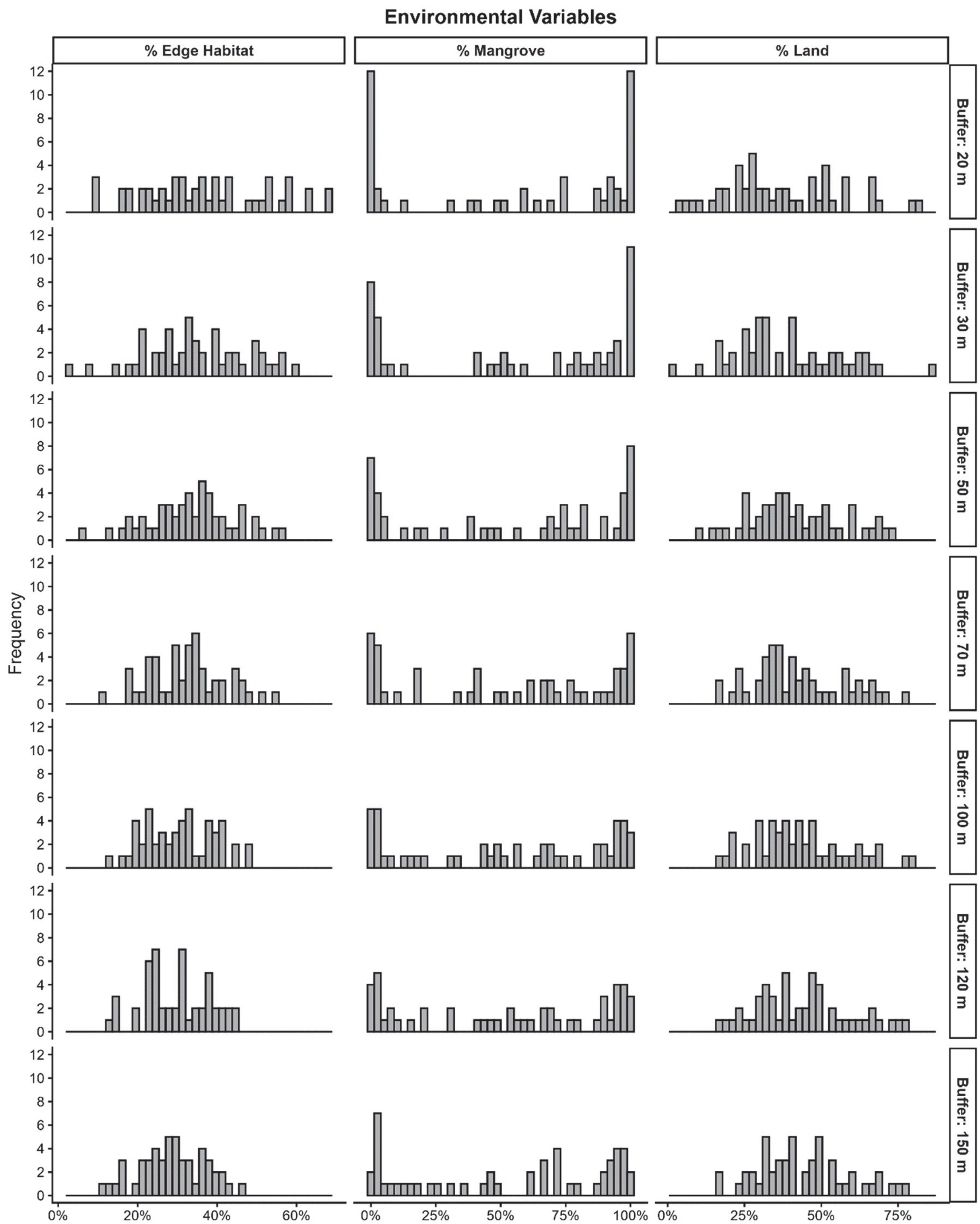

Figure S3. Histograms of habitat variables from all 52 sampling sites calculated at each candidate scale of effect using drone-based habitat imagery. Sites were chosen based on spatially stratified random sampling to optimize for mangrove cover at 200 meters. Columns are the three habitat variables we used, and each row is a candidate scale used in the modeling workflow. Metrics were calculated with edge defined as land within three meters of the water's edge.

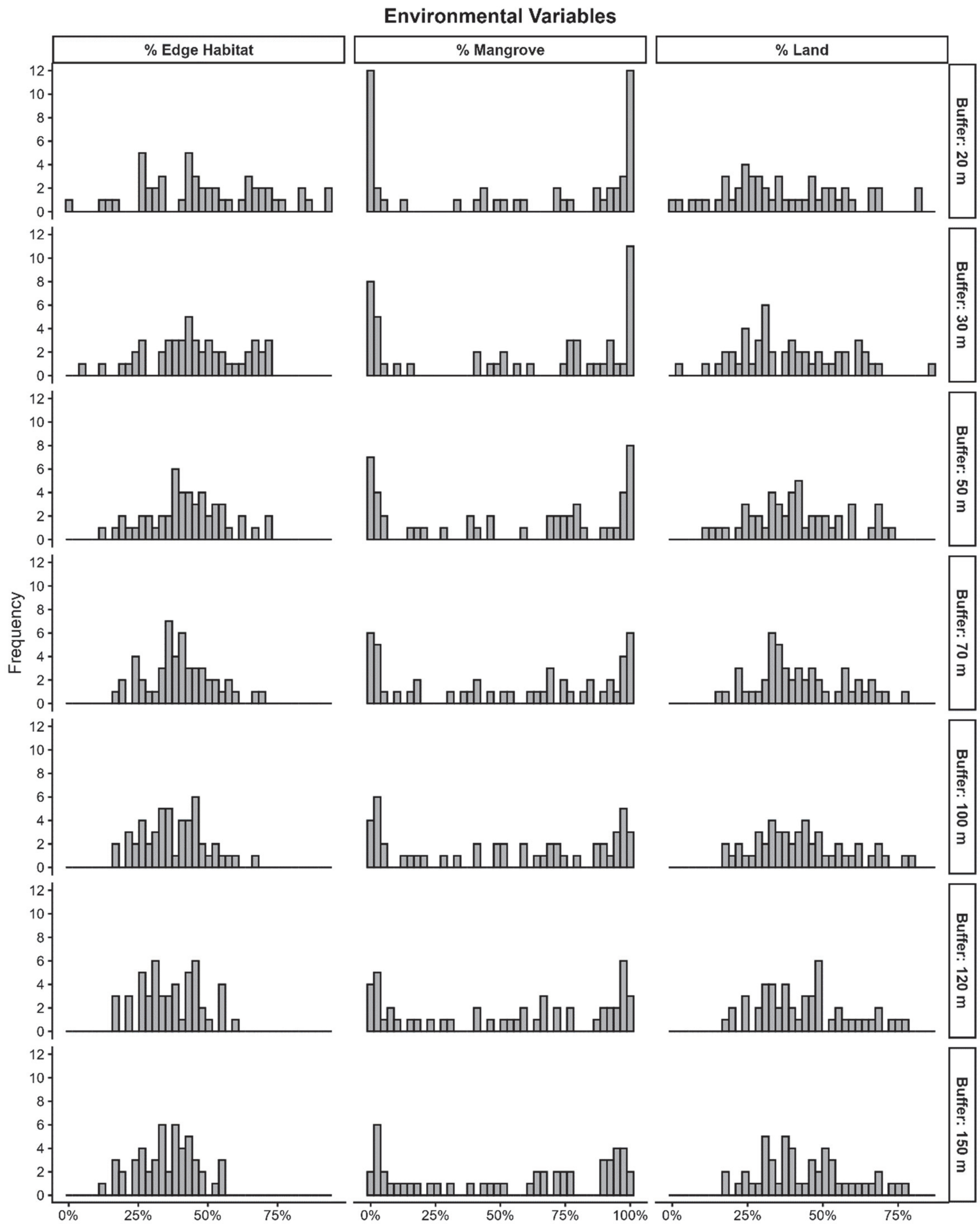

Figure S4. Histograms of habitat variables from all 52 sampling sites calculated at each candidate scale of effect using drone-based habitat imagery. Sites were chosen based on spatially stratified random sampling to optimize for mangrove cover at 200 meters. Columns are the three habitat variables we used, and each row is a candidate scale used in the modeling workflow. Metrics were calculated with edge defined as land within five meters of the water's edge.

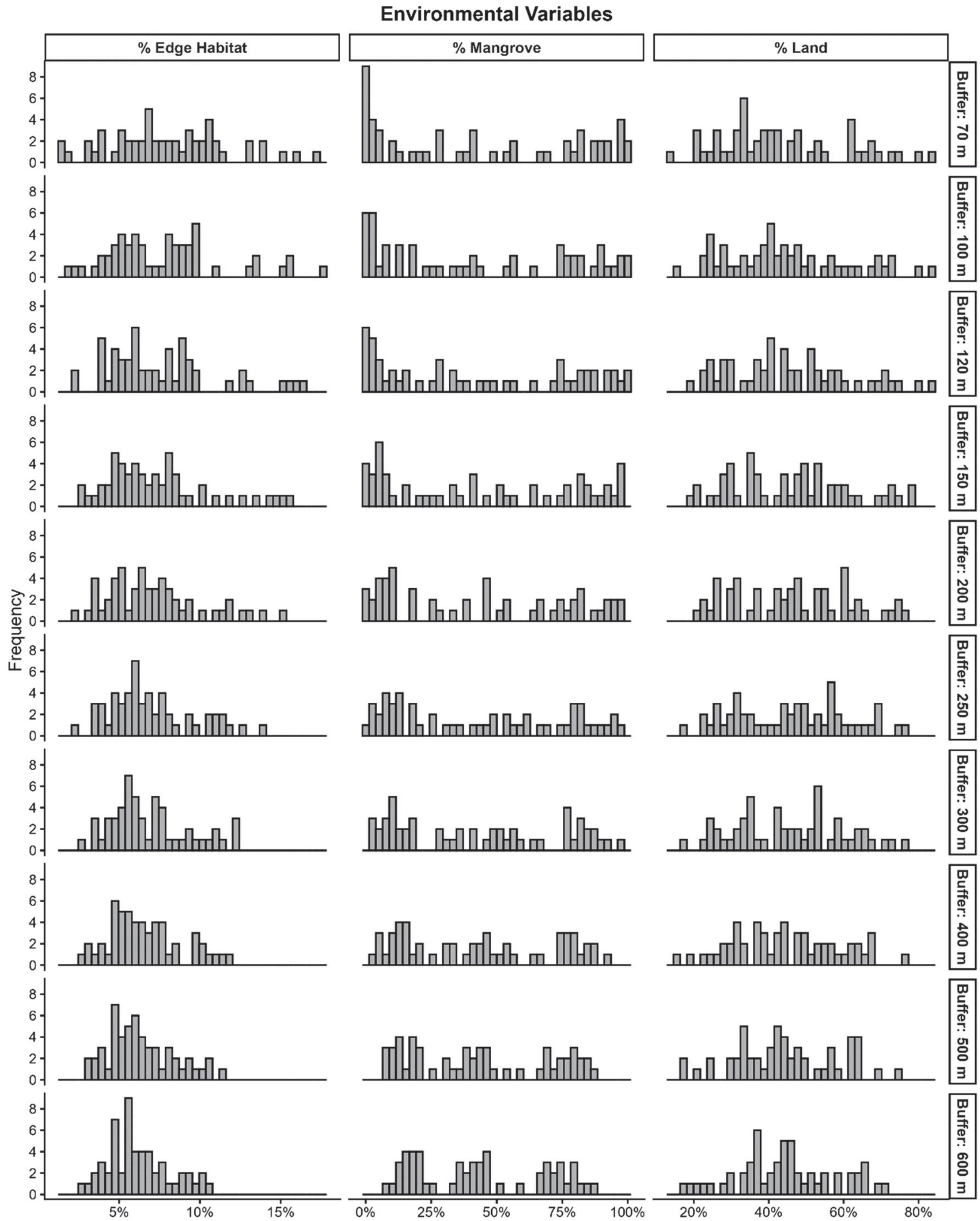

Figure S5. Histograms of habitat variables from all 52 sampling sites calculated at each candidate scale of effect using satellite-based habitat imagery. Sites were chosen based on spatially stratified random sampling to optimize for mangrove cover at 200 meters. Columns are the three habitat variables we used, and each row is a candidate scale used in the modeling workflow. Metrics were calculated with edge defined as land within one meter of the water's edge.

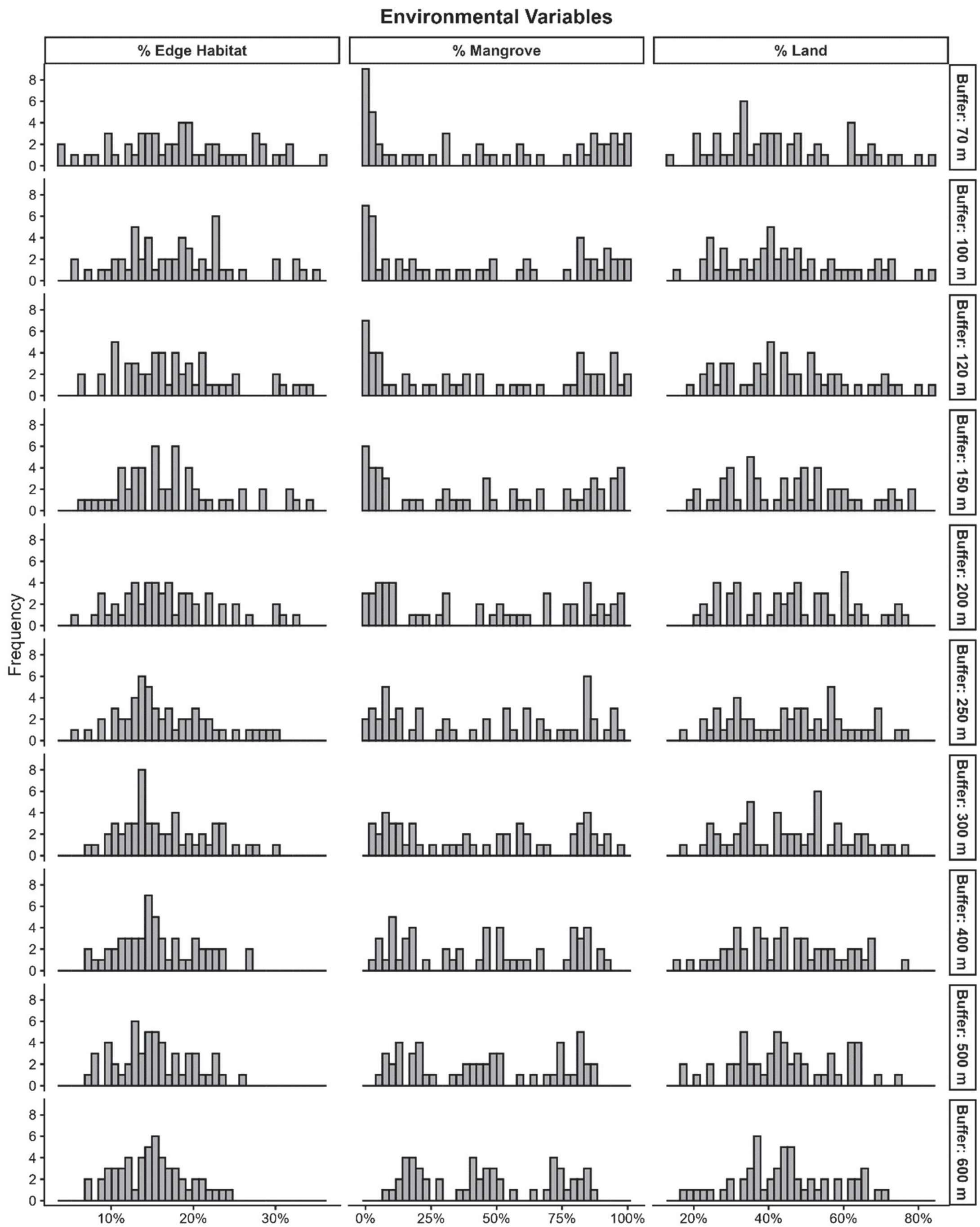

Figure S6. Histograms of habitat variables from all 52 sampling sites calculated at each candidate scale of effect using satellite-based habitat imagery. Sites were chosen based on spatially stratified random sampling to optimize for mangrove cover at 200 meters. Columns are the three habitat variables we used, and each row is a candidate scale used in the modeling workflow. Metrics were calculated with edge defined as land within three meters of the water's edge.

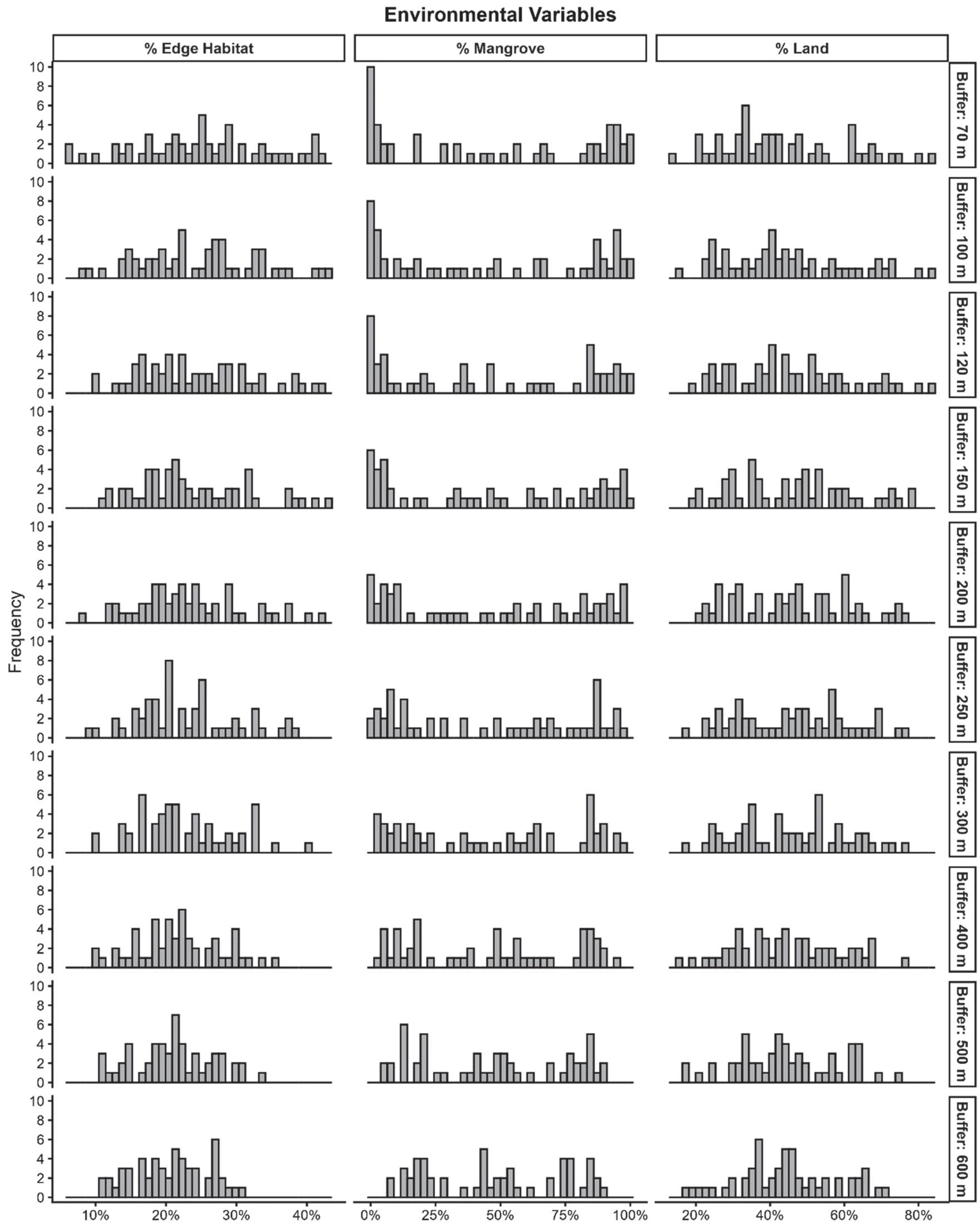

Figure S7. Histograms of habitat variables from all 52 sampling sites calculated at each candidate scale of effect using satellite-based habitat imagery. Sites were chosen based on spatially stratified random sampling to optimize for mangrove cover at 200 meters. Columns are the three habitat variables we used, and each row is a candidate scale used in the modeling workflow. Metrics were calculated with edge defined as land within five meters of the water's edge.

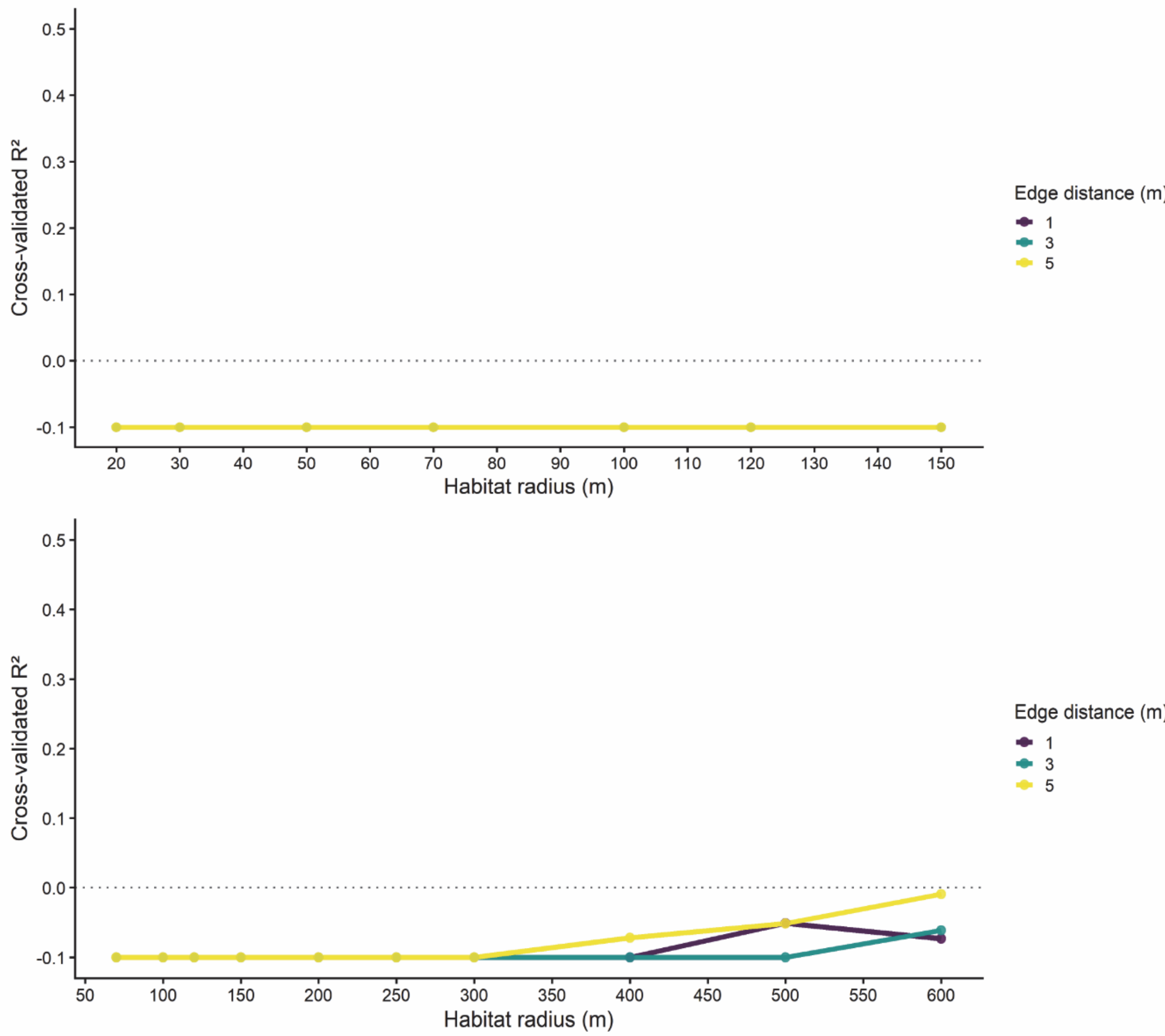

Figure S8. Cross validated R2 values for GAM models of grass shrimp abundance over spatial scales. The drone data analysis (top-plot) used high-resolution spatial data from drone flights covering smaller spatial scales from 20 – 150m around the sampling site. The satellite-based data analysis used courser resolution but could capture a greater spatial extent from 70 – 600m around the sampling site. No model across any scale showed any ability to reliably predict out-of-sample data, and the cross validated R^2 value was less than zero for all scales.

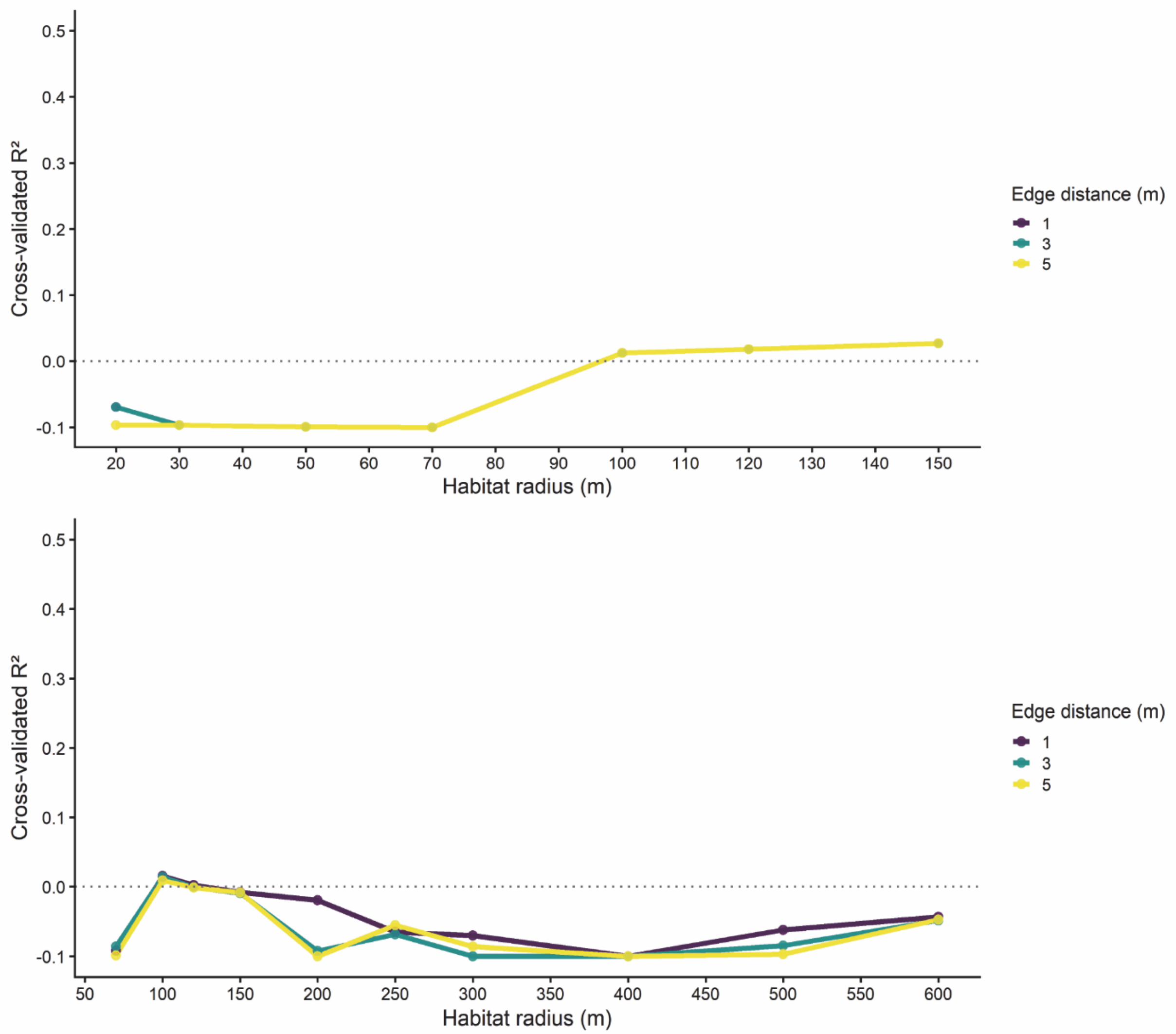

Figure S9. Cross validated R2 values for GAM models of darter goby abundance over spatial scales. The drone data analysis (top-plot) used high-resolution spatial data from drone flights covering smaller spatial scales from 20 – 150m around the sampling site. The satellite-based data analysis used courser resolution but could capture a greater spatial extent from 70 – 600m around the sampling site. The drone-based model included only % Land, which did not vary by edge distance. All models had very low out-of-sample predictive accuracy.

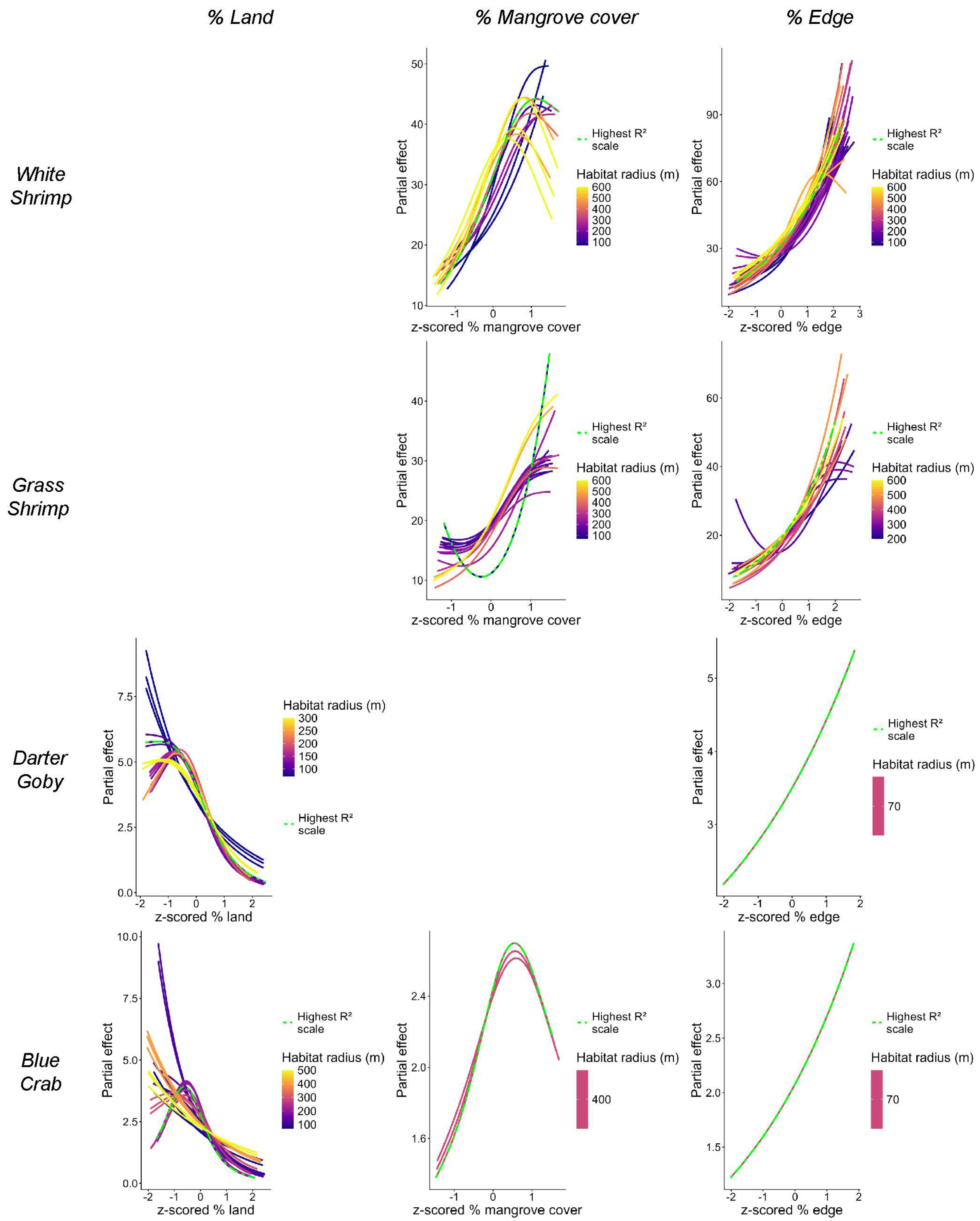

Figure S10. Partial effect plots for the selected GAM model for each species at the satellite-scale analysis. Habitat variables are standardized for all scales. Plots in the same row correspond to a single species, and columns correspond to a particular variable. The partial effect curve for model with the highest R^2 value is highlighted in each plot. Spaces are blank when the column variable was not significant at any scale in the model for the row's species for the satellite scale analysis.

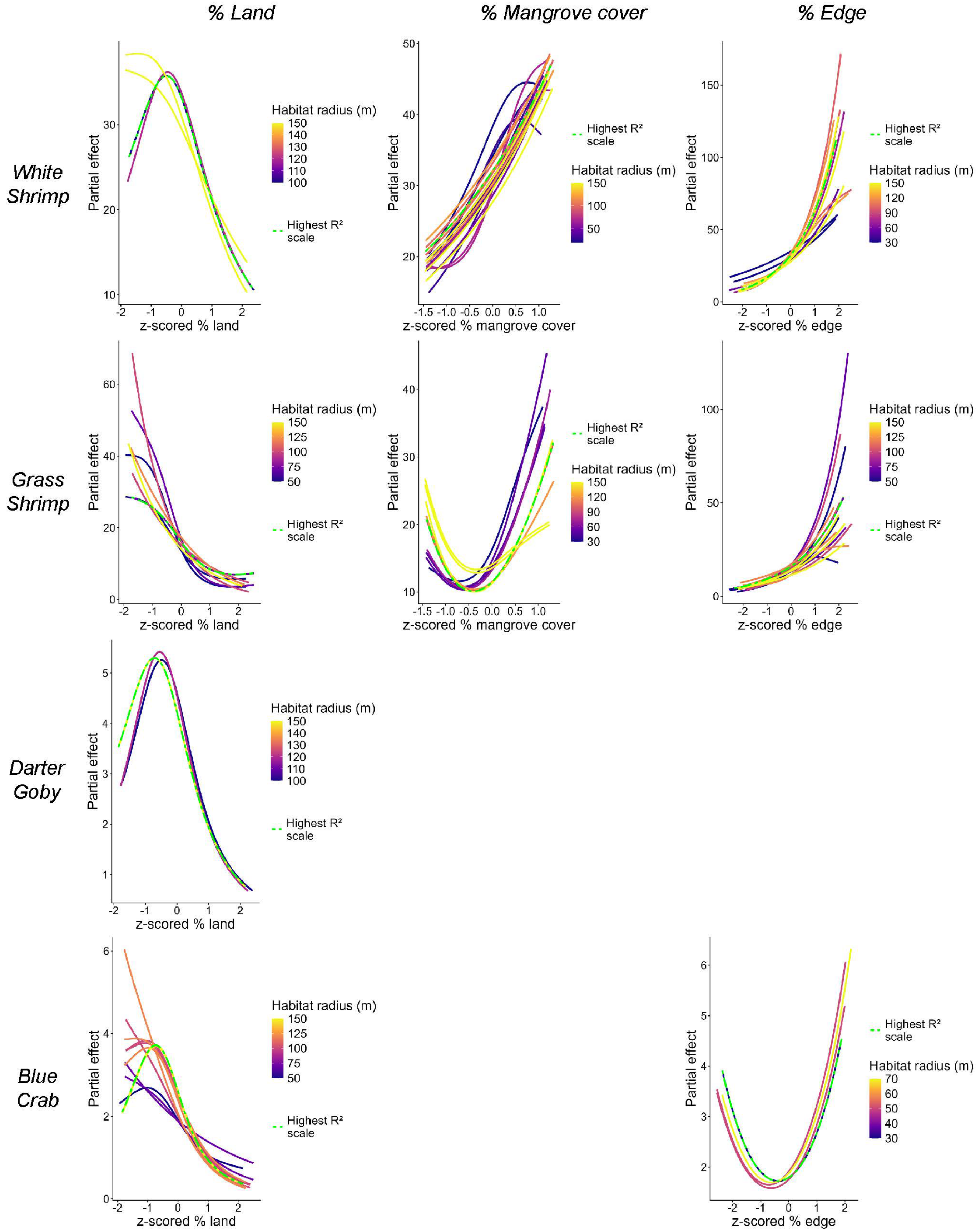

Figure S11. Partial effect plots for the selected GAM model for each species at the drone-scale analysis. Habitat variables are standardized for all scales. Plots in the same row correspond to a single species, and columns correspond to a particular variable. The partial effect curve for model with the highest R2 value

is highlighted in each plot. Spaces are blank when the column variable was not significant at any scale in the model for the row's species for the drone scale analysis.
